# Layer-wise relevance propagation of InteractionNet explains protein–ligand interactions at the atom level

**DOI:** 10.1038/s41598-020-78169-6

**Published:** 2020-12-03

**Authors:** Hyeoncheol Cho, Eok Kyun Lee, Insung S. Choi

**Affiliations:** Department of Chemistry, KAIST, Daejeon, 34141 Korea

**Keywords:** Computer science, Cheminformatics, Physical chemistry

## Abstract

Development of deep-learning models for intermolecular noncovalent (NC) interactions between proteins and ligands has great potential in the chemical and pharmaceutical tasks, including structure–activity relationship and drug design. It still remains an open question how to convert the three-dimensional, structural information of a protein–ligand complex into a graph representation in the graph neural networks (GNNs). It is also difficult to know whether a trained GNN model learns the NC interactions properly. Herein, we propose a GNN architecture that learns two distinct graphs—one for the intramolecular covalent bonds in a protein and a ligand, and the other for the intermolecular NC interactions between the protein and the ligand—separately by the corresponding covalent and NC convolutional layers. The graph separation has some advantages, such as independent evaluation on the contribution of each convolutional step to the prediction of dissociation constants, and facile analysis of graph-building strategies for the NC interactions. In addition to its prediction performance that is comparable to that of a state-of-the art model, the analysis with an explainability strategy of layer-wise relevance propagation shows that our model successfully predicts the important characteristics of the NC interactions, especially in the aspect of hydrogen bonding, in the chemical interpretation of protein–ligand binding.

## Introduction

The approach of deep learning has recently been adopted to the chemistry discipline for tackling diverse chemical tasks, such as prediction of physicochemical properties, protein–ligand interactions, and retrosynthetic analysis^1–4,11–14^. Human-curated heuristics and descriptors have been used for decades in cheminformatics including early machine learning methods, and it is the representation learning of three-dimensional molecules that is one of the recent endeavors of deep-learning chemistry. The direct learning of molecular structures for the prediction of target properties, without prior assessment on the structures or quantum-chemical calculations, has been enabled by the remarkable discriminative ability of deep neural networks (DNNs)^5,6^. Compared with the physics-based computational methods for calculating molecular properties, the deep-learning approach offers a fast, but still powerful, option for estimating diverse characteristics of molecules through the data-driven discovery of molecular patterns^7,8^.

The recent rise of graph neural networks (GNNs) has upscaled deep-learning capability in chemistry with the easy handling of molecules as molecular graphs, which are defined by two sets of vertices and edges^9,10^. The molecular graphs contain the structural information on molecules in two-dimensional (2D) space, with atoms as vertices and bonds as edges in the graphs. In the GNN, the neurons in a layer are connected to their graph neighborhoods, and layer stacking generates broader local structures in molecules. Many GNN models have been developed for the prediction of molecular energies^7,8^, physical properties^11,12^, protein interactions^13,14^, and biochemical functions^15,16^. Since the pioneering report by Baskin et al.^17^ on the utilization of molecular graphs for the prediction of physicochemical properties of hydrocarbons and other molecules, Duvenaud et al.^11^ and Kearnes et al.^15^ have examined the GNN approaches on the prediction of molecular properties, and the GNN architecture has further been refined to message-passing neural networks (MPNNs) that outperform other machine-learning methods based on molecular fingerprints^7^. Moreover, the expansion of the GNN architecture into the 3D space for modeling the actual molecular structures has recently been explored, and the efficacy of the GNN approach on the problems requiring 3D molecular structures has been proven^14,18^.

One of the focused fields of deep learning in chemistry is the replacement of the scoring function on the structure-based drug design with data-driven DNN models^13,14,19–23^. The essence of DNN models for deep-learning scoring compared to the force field energy functions and scoring functions is the appropriate database to learn molecular patterns and their relationship to binding affinity, and they are strongly linked to prediction performance. The PDBbind database^24,25^ is the most widely used dataset, which is a curation of 3D protein–ligand structures obtained from X-ray crystallography and multidimensional NMR techniques with complementary binding affinities, for training DNN models on prediction of the binding constant from a complex structure. Many DNN models were developed based on the PDBbind database and can be classified into two categories: convolutional neural networks (CNNs) with voxelized images and GNNs working on graph representations of complexes.

Rapid development of high-performance and deeply-stacked CNN models in computer science has dramatically raised the prediction performance of binding constants through enhanced pattern recognition of the 3D molecular images^22,23,26,27^. Protein–ligand complexes were transferred into the angstrom-level voxel grid and used for training the CNN models. Meanwhile, the GNN models^13,14^, which focused on the interpretation of molecular bonding (i.e., covalent bonds) as graph edges, was utilized after the success of CNN models by incorporating noncovalent (NC) interactions as graph edges in molecular graphs, which play significant roles in the programmed formation of 3D molecular structures of biomolecules (e.g., proteins, nucleic acids, and lipid bilayers) and polymers and their dynamics^28–30^. In the GNN models, NC connectivity was utilized in conjunction with covalent connectivity for post-refinement of atomic features after the convolution with covalent-bond connectivity. Gaussian decay functions have been used to mimic decreased influences from distant atoms, or multiple kernels for distance bins have been adopted for simulating NC interactions^8,13,31^. These approaches enrich the graphic representation of molecules by adding topological information and acquiring the shape-awareness, which has only been feasible in the CNNs. In addition to the decay simulation, an approximation of the entire atomic contribution to a smaller subset was widely utilized^14,20–22^. Due to the extremely large number of atoms in the protein–ligand complex compared to other molecules in molecular property datasets, training the complex data is challenging for both CNN and GNN models. By limiting the protein atoms into a spatial neighborhood of the ligand molecule, the complex can be greatly reduced into smaller sizes and trained efficiently without losing important interactions. Owing to the aforementioned advanced approaches, the GNN models became a competitive option for developing deep-learning scoring models with a direct interpretation of molecular structures.

In this paper, we propose a GNN architecture, denoted as InteractionNet, that directly learns molecular graphs without any physical parameters, wherein the NC interactions are encoded as graphs along with the bonded adjacency that models covalent interactions. We utilize the PDBbind dataset for evaluation of the concept and examine the model performance on predicting the binding constant from a complex structure. Specifically, we divide the convolutional layers in InteractionNet into two, the covalent and NC convolution layers (CV_[C]_ and CV_[NC]_ layers, respectively), and evaluate the significance of NC convolution. There have been reports on the incorporation of NC connectivity in GNN models, but in strict combination with covalent connectivity. Here, we apply the covalent and NC connectivity separately to investigate the importance of each convolution layer, which has not been explored. In extreme cases, only CV_[NC]_ is used, without any CV_[C]_ layers, and compared to other models. Moreover, we investigate the optimal cropping strategy for downsizing the protein–ligand structure and efficient training. Based on the findings, we further investigate the explanations for the predictions of the trained model, i.e., how the trained model predicts for the first time from the given input data in the protein–ligand complexation problem. By performing decomposition-based, layer-wise relevance propagation (LRP)^32,33^ on behalf of explainable AI^34–36^ and visualizing the obtained atomic contribution for the prediction of the protein–ligand complex, we explore the relationship between machine-predicted NC interactions and knowledge-based NC interactions from the molecular structures.

## Results and discussion

### InteractionNet architecture

**Figure 1:**
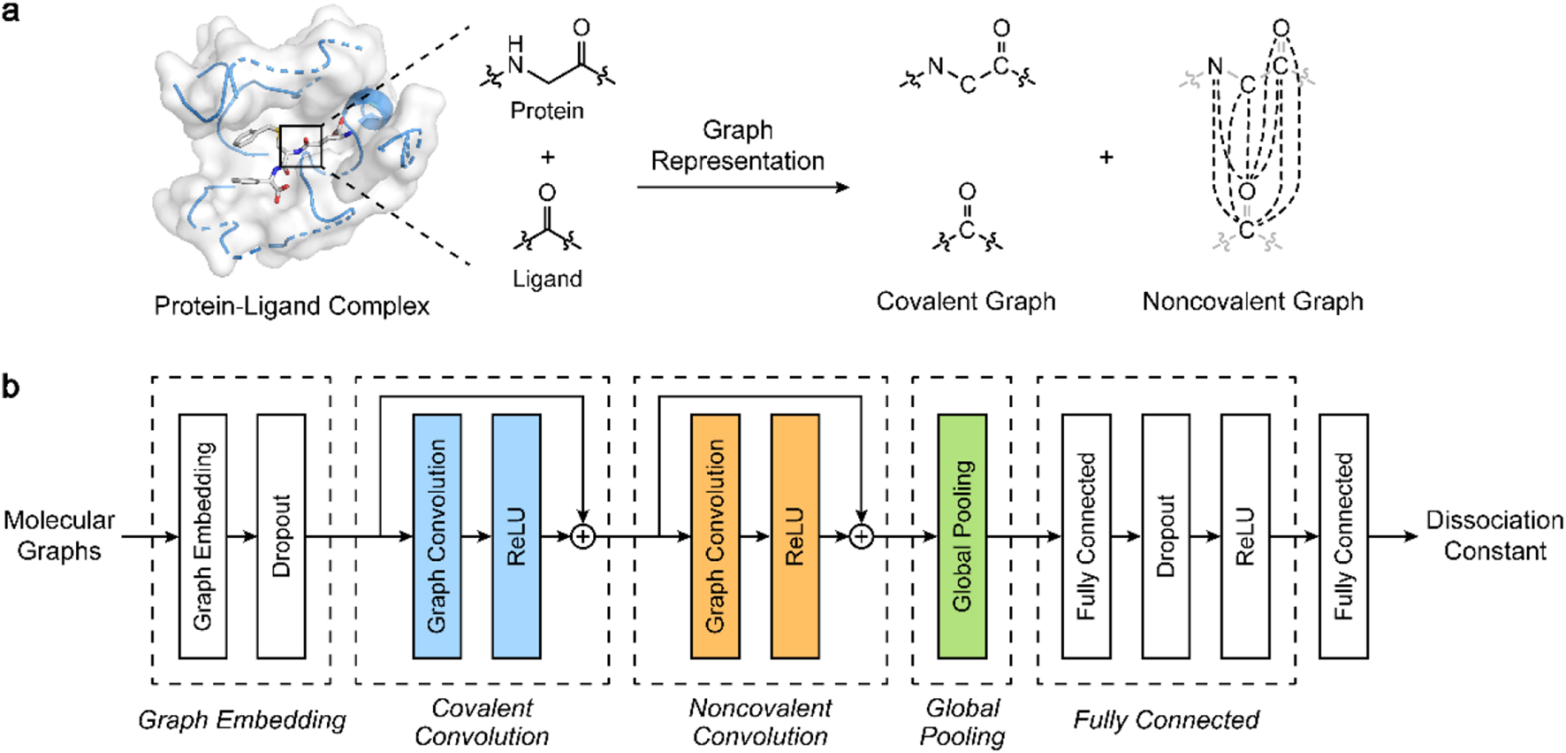
(**a**) Schematic illustrations for modeling NC interactions within a graphic representation, and (**b**) architecture of InteractionNet for predicting the dissociation constant from the covalent and NC graphs. (**a**) Structure of the protein–ligand complex converted into two graph representations, encoded by covalent ($\mathrm{A}$_[C]_) and NC adjacency matrices ($\mathrm{A}$_[NC]_), defined by covalent bond connectivity and all possible edges between the protein and the ligand, respectively. (**b**) InteractionNet learns the two aforementioned adjacency matrices, $\mathrm{A}$_[C]_ and $\mathrm{A}$_[NC]_, and predicts the dissociation constant of the complex through a graphic neural network consisting of five functional layers.

For graphic representation of a protein–ligand complex, InteractionNet employs two adjacency matrices for the complex, denoted the covalent and NC adjacency matrices ($\mathrm{A}$_[C]_ and $\mathrm{A}$_[NC]_), similar to the PotentialNet reported by Feinberg et al.^13^ $\mathrm{A}$_[C]_ and $\mathrm{A}$_[NC]_ are defined by the combination of molecular graphs for a protein and a ligand but with different connectivity strategies. The covalent adjacency matrix, $\mathrm{\rm A}$_[C]_, consists of the bond connectivity in the protein and the ligand, and is constructed by a disjoint union of the protein and the ligand graphs, maintaining the bond connectivity only within each molecule. The NC adjacency matrix, $\mathrm{\rm A}$_[NC]_, defined by a graph having full connectivity between the vertices of the protein and the ligand graphs, contains all the possible edges between the protein and the ligand but not within the same molecule (Fig. 1a). Based on the notation used by Feinberg et al.^13^, each adjacency matrix can be decomposed into four blocks, ${\mathrm{A}}_{L:L}$, ${\mathrm{A}}_{L:P}$, ${\mathrm{A}}_{P:L}$, and ${\mathrm{A}}_{P:P}$, that correspond to smaller adjacency matrices encoding the connectivity between ligand–ligand, ligand–protein, protein–ligand, and protein–protein atoms, respectively (Eq. 1).

1 $$\mathrm{A}=\left[\begin{array}{ccc}{\mathrm{A}}_{11}& \cdots & {\mathrm{A}}_{1N}\\ \vdots & \ddots & \vdots \\ {\mathrm{A}}_{N1}& \cdots & {\mathrm{A}}_{NN}\end{array}\right]=\left[\begin{array}{cc}{\mathrm{A}}_{L:L}& {\mathrm{A}}_{L:P}\\ {\mathrm{A}}_{P:L}& {\mathrm{A}}_{P:P}\end{array}\right]$$

where ${\mathrm{A}}_{ij}$ is whether the node $i$ and $j$ are adjacent, and $N$ is the number of atoms inside the complex. For ${\mathrm{ A}}$_[C]_, only the ${\mathrm{A}}_{L:L}$ and ${\mathrm{A}}_{P:P}$ blocks are filled with the existence of a covalent bond between atoms, and the remaining ${\mathrm{A}}_{L:P}$ and ${\mathrm{A}}_{P:L}$ blocks are filled with 0. In the case of $\mathrm{\rm A}$_[NC]_, ${\mathrm{A}}_{L:P}$ and ${\mathrm{A}}_{P:L}$ blocks are filled with 1, implying all possible NC interactions between atoms, and the rest are filled with 0. These adjacency strategies assume that there is no covalent bond between the protein and the ligand, and NC interactions within the same molecule are ignored. Obtained adjacency matrices are used as-is through the neural network without training the adjacency matrix itself or requiring modification during the propagation.

InteractionNet is built to utilize the $\mathrm{A}$_[C]_ and $\mathrm{A}$_[NC],_ consecutively, for the end-to-end prediction of dissociation constants from the molecular structures (Fig. 1b). It consists of five functional layers: node-embedding layers, CV_[C]_ layers, CV_[NC]_ layers, a global pooling (GP) layer, and fully-connected (FC) layers. The node-embedding layers update the atomic feature matrix $\mathrm{X}$ into ${\mathrm{X}}_{\mathrm{NE}}$ through fully-connected neural networks that mix the features assigned for each atom (Eq. 2). The CV_[C]_ and CV_[NC]_ layers receive the node-embedded ${\mathrm{X}}_{\mathrm{NE}}$ and combine the graph adjacency with node embedding for local aggregation of the information. Compared with the PotentialNet^13^, we separate the graph convolution layers utilizing the two adjacency matrices, $\mathrm{A}$_[C]_ and $\mathrm{A}$_[NC]_, one-by-one at each layer (Eqs. 3, 4), whereas they are combined and utilized in a single layer in the PotentialNet. By applying the two adjacency matrices for the graph convolution separately, we simulate the importance of each step on the prediction of the dissociation constant independently. For each convolution step, InteractionNet utilizes the corresponding adjacency matrix and updates the representation additively by residual connections^26^. After the convolution steps, the GP layer aggregates the atomic features distributed across the atoms in a permutation-invariant way and generates a molecular vector. Sum pooling was utilized for the GP mechanism to aggregate atomic feature matrix ${\mathrm{X}}_{\mathrm{NC}}$ obtained from the convolutions into molecular vector ${\mathrm{X}}_{\mathrm{GP}}$ (Eq. 5). With the obtained molecular vector ${\mathrm{X}}_{\mathrm{GP}}$, the FC layers transform the representation into the dissociation constant of a protein–ligand complex (Eq. 6).

2 $${\mathrm{X}}_{\mathrm{NE}}=\mathrm{ReLU}\left(\mathrm{ReLU}\left({\mathrm{XW}}_{\mathrm{NE},1}+{\mathrm{b}}_{\mathrm{NE},1}\right){\mathrm{W}}_{\mathrm{NE},2}+{\mathrm{b}}_{\mathrm{NE},2}\right)$$

3 $${\mathrm{X}}_{\mathrm{C}}={\mathrm{X}}_{\mathrm{NE}}+\mathrm{ReLU}\left({\mathrm{A}}_{[\mathrm{C}]}{\mathrm{X}}_{\mathrm{NE}}{\mathrm{W}}_{\mathrm{C}}\right)$$

4 $${\mathrm{X}}_{\mathrm{NC}}={\mathrm{X}}_{\mathrm{C}}+\mathrm{ReLU}\left({\mathrm{A}}_{\left[\mathrm{NC}\right]}{\mathrm{X}}_{\mathrm{C}}{\mathrm{W}}_{\mathrm{NC}}\right)$$

5 $${\mathrm{X}}_{\mathrm{GP}}={1}^{\mathrm{T}}{\mathrm{X}}_{\mathrm{NC}}$$

6 $${\mathrm{X}}_{\mathrm{NC}}=\mathrm{ReLU}\left(\mathrm{ReLU}\left({\mathrm{X}}_{\mathrm{GP}}{\mathrm{W}}_{\mathrm{FC},1}+{\mathrm{b}}_{\mathrm{FC},1}\right){\mathrm{W}}_{\mathrm{FC},2}+{\mathrm{b}}_{\mathrm{FC},2}\right)$$

where $\mathrm{W}$ and $\mathrm{b}$ represent the trainable weight matrix and the bias in each linear combinations, respectively. $\mathrm{ReLU}$ is the rectified linear unit, and $1$ denotes all-ones vector.

### Model training

We examined the efficacy of the CV_[C]_ and CV_[NC]_ layers by three variants of InteractionNet with different compositions of CV layers. Four other functional layers of InteractionNet were used with the same number of layers and composition across the variants. By incorporating only one type of the CV layers for InteractionNet, we built InteractionNet_[C],_ utilizing only CV_[C]_ layers, and InteractionNet_[NC],_ utilizing only CV_[NC]_ layers. The variant that incorporated both CV layers sequentially was coined as InteractionNet_[C-NC]_. In the chemists’ points of view, InteractionNet_[C]_ focused on the covalent bonds within each ligand and protein molecule for prediction, InteractionNet_[NC]_ did this on NC interactions between the ligand and the protein, and InteractionNet_[C-NC]_ observed covalent bonds first and then used the generated information for the secondary refinement through NC interactions. We chose the dissociation constant of the protein–ligand complex (*K*_d_) as our prediction target because the protein–ligand binding is governed primarily by the NC interactions, not covalent bonds, which is important in investigating the efficacy of the proposed architecture. We conducted a 20-fold-cross-validated experiment on the refined set of the PDBbind v2018 dataset^24,25^, consisting of 4186 complexes and their experimental *K*_d_ values.

In the data preprocessing for model training, we cropped the protein structure for faster training and less memory consumption. The binding pockets of the proteins in the refined PDBbind set contained a maximum of 418 atoms, which was almost 16 times larger than the ligands that had only 26 atoms at maximum. We thought that the interactions between a protein and a ligand could be simulated with a smaller subset of atoms in the protein because the number of atoms that participated in the protein–ligand binding is much less than the maximum value. The appropriate cropping strategy, without any loss in performance, is also highly important for efficient training, considering the exponential increase in the memory consumption of the training data. We utilized the spatial atom filtering for simplification of protein structures, which excluded the atoms of a protein distant from a ligand by the range cutoff. In detail, the shortest distance of a protein atom to the ligand atoms was measured, and the protein atom was excluded if the distance exceeded the predefined range cutoff. By spatial cropping with the range cutoff, we obtained a subset of the protein structures, similar to the shape of the van der Waals surface of the ligand but with a much larger radius, and used the subset for the generation of the molecular graphs.

**Figure 2:**
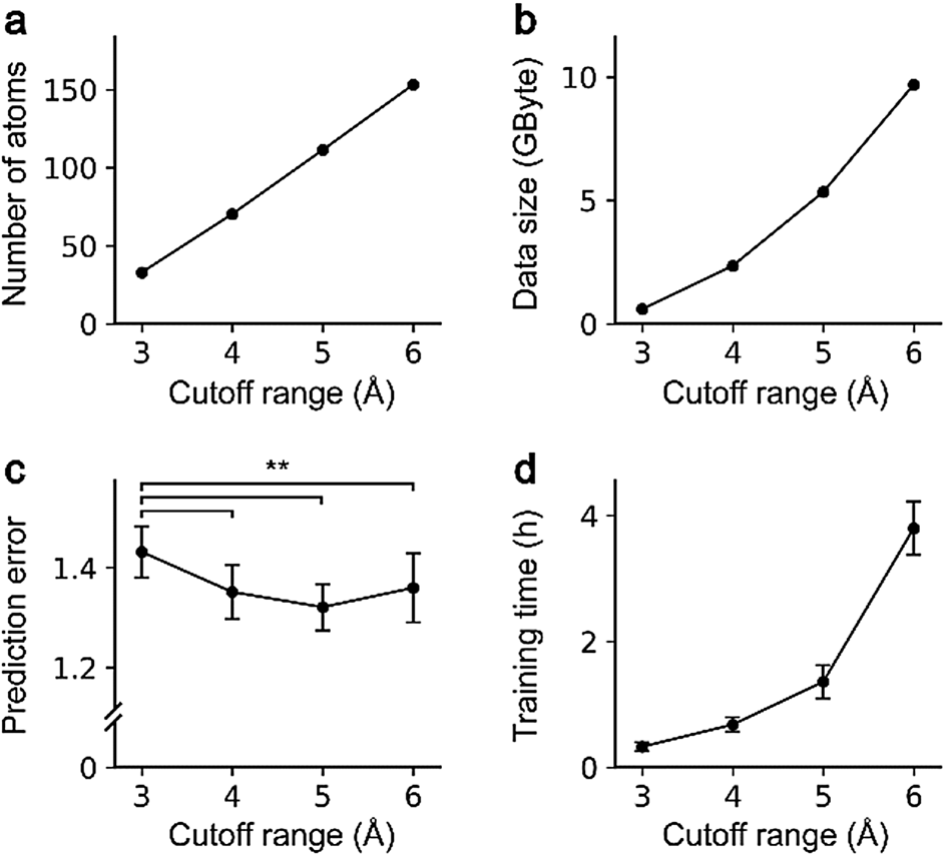
Influence of the protein cutoff range from 3 to 6 Å on (**a**) the average number of atoms included in a complex, (**b**) the size of the training data, (**c**) the root-mean-squared-error for the predictions from the trained model, and (**d**) the average single-fold training time. Error bars indicate standard deviations for each measurement. **p < 0.005.

For investigating the influence of the cutoff applied to crop the protein structure into the ligand neighborhoods, we compared the averaged model performance and the training time using InteractionNet_[C-NC]_ by changing the cutoff with 1-Å increment. The number of atoms included in the cropped complex increased linearly with respect to the cutoff, while the data size for the training dataset increased exponentially (Fig. 2a,b). The averaged performance was saturated from 4 Å, confirmed by the one-way analysis of variance (ANOVA) with the posthoc Tukey HSD test (Fig. 2c). The corresponding training time increased dramatically as the cutoff increased (Fig. 2d). Based on the observation, the 5-Å cutoff was considered the most appropriate for our system and used for further investigations.

### Prediction of dissociation constants

The root-mean-square error (RMSE) results from the cross-validation experiments, based on the 5-Å filtering of protein structures, confirmed that the CV_[NC]_ layers played a significant role in the *K*_d_ prediction from the molecular graphs (Table 1). InteractionNet_[C-NC]_ and InteractionNet_[NC]_ outperformed InteractionNet_[C]_, regardless of the number of CV layers. For example, the RMSE values for InteractionNet_[C-NC]_ and InteractionNet_[C]_ were 1.321 and 1.379, respectively, showing a 4% improvement by incorporating CV_[NC]_ layers (*p* < 0.005; one-way ANOVA with the posthoc Tukey HSD test). The performance of InteractionNet_[C-NC]_ was measured to be slightly higher than InteractionNet_[NC]_, but the difference was not significant in statistical analysis (*p* = 0.450). These results indicated that the interactions between a protein and a ligand could be simulated accurately, even with a single CV_[NC]_ layer, without any help from previous covalent-refinement steps. We compared the performance of InteractionNet with that of PotentialNet, which was the state-of-the-art GNN model for prediction of protein–ligand affinity. For the comparison, we built an in-house PotentialNet model according to the original paper^13^. In detail, we replaced the CV_[C]_ and CV_[NC]_ layers of InteractionNet with the stages 1 and 2 of PotentialNet, respectively, and trained the in-house PotentialNet model with the same dataset used in InteractionNet. The averaged RMSE value for the test set was measured to be 1.343 in the case of the PotentialNet, which was comparable to that of InteractionNet (statistically insignificant; one-way ANOVA with the posthoc Tukey HSD test).

**Table 1:** Twenty-fold cross-validation results for InteractionNet on the refined set of the PDBbind v2018.

Model	Train	Validation	Test
InteractionNet_[C]_	1.115 ± 0.085	1.355 ± 0.060	1.379 ± 0.057
InteractionNet_[NC]_	1.035 ± 0.077	1.328 ± 0.042	1.340 ± 0.044
InteractionNet_[C-NC]_	**0.950 ± 0.032**	1.313 ± 0.107	**1.321 ± 0.045**
PotentialNet	0.956 ± 0.105	**1.307 ± 0.054**	1.343 ± 0.037

Root-mean-square-errors were measured for each trial and averaged (mean ± standard deviation). The best results were highlighted in boldface.

**Figure 3:**
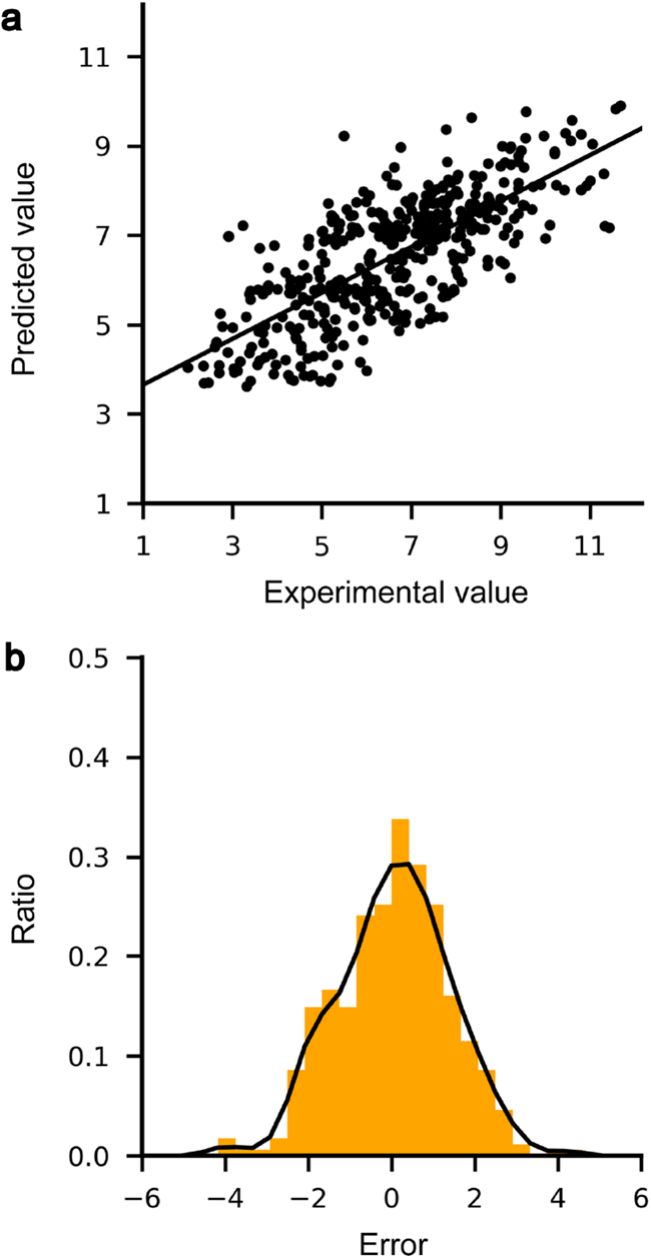
(**a**) Scatterplot and (**b**) error distribution of predicted and experimental *K*_d_ values for 419 complexes included in the test set. (**a**) The scatterplot for predicted versus experimental *K*_d_ is depicted with the trend line (a solid line). (**b**) The error histogram (orange) and distribution (black) for predictions from the test set. The most similar trial in performance to the average was selected for depicting the graphs in the cross-validation trials.

For visualization of the prediction trends of the model, we selected the cross-validation trial, the measured RMSE value of which was most similar to the average of 20 repetitive trials, and used the test set in the selected trial as a representative set for obtaining a scatterplot and an error histogram. The scatterplot for the predicted *K*_d_ values revealed a high correlation with the experimental *K*_d_ in a linear relationship (Fig. 3a), and the error distribution showed a Gaussian-like, zero-centered shape (Fig. 3b). It is to be noted that 20 cross-validation trials showed similar trends in the scatterplot and the error histogram, but had small differences in pattern (Fig. S1).

### Layer-wise relevance propagation (LRP)

**Figure 4:**
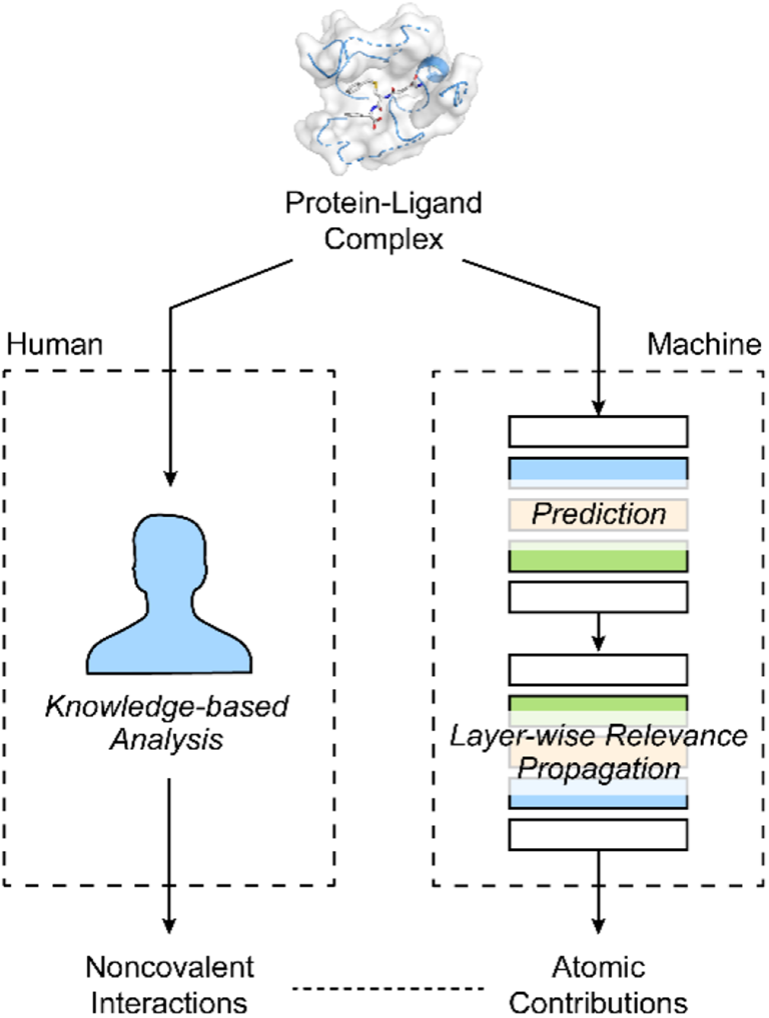
Schematic illustration of atomic contributions obtained by applying layer-wise relevance propagation (LRP) on InteractionNet and its comparison with knowledge-based protein–ligand interactions.

**Figure 5:**
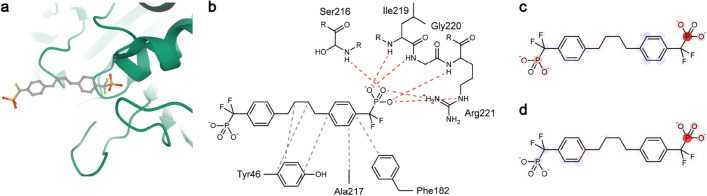
(**a**) Three-dimensional structure of the protein–ligand complex, 1KAV. The protein is depicted in a cartoon (green), and the ligand is depicted in color-coded ball-and-stick. Atom colors: gray (carbon), red (oxygen), orange (phosphorus), and light green (fluorine). (**b**) Knowledge-based estimation of protein–ligand interactions. Hydrogen bonds are depicted in red dashed lines, and hydrophobic contacts are depicted in gray dashed lines. (**c**) Heat map for the atomic contributions on the *K*_d_ prediction, obtained from the LRP on PotentialNet. (**d**) Heat map for the atomic contributions on the *K*_d_ prediction, obtained from the LRP on InteractionNet. The contributions are illustrated with color intensity of red (positive influence), white (zero influence), and blue (negative influence) colors.

**Figure 6:**
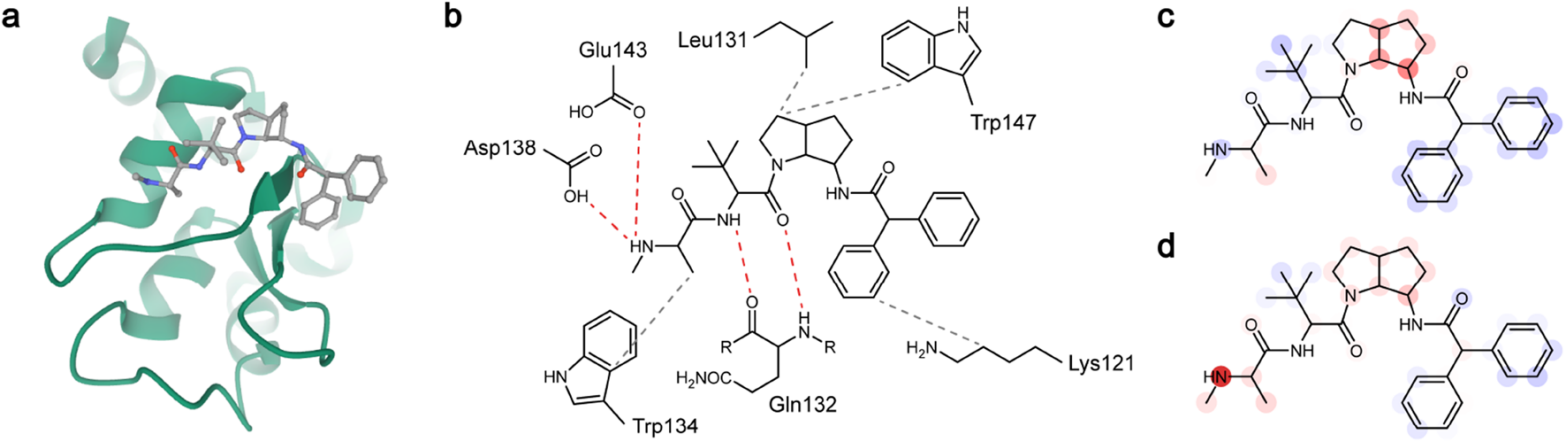
(**a**) Three-dimensional structure of the protein–ligand complex, 3F7H. The protein is depicted in a cartoon (green), and the ligand is depicted in color-coded ball-and-stick. Atom colors: gray (carbon), red (oxygen), and blue (nitrogen). (**b**) Knowledge-based estimation of protein–ligand interactions. Hydrogen bonds are depicted in red dashed lines, and hydrophobic contacts are depicted in gray dashed lines. (**c**) Heat map for the atomic contributions on the prediction of *K*_d_, obtained from the LRP on PotentialNet. (**d**) Heat map for the atomic contributions on the prediction of *K*_d_, obtained from the LRP on InteractionNet. The contributions are illustrated with color intensity of red (positive influence), white (zero influence), and blue (negative influence) colors.

**Figure 7:**
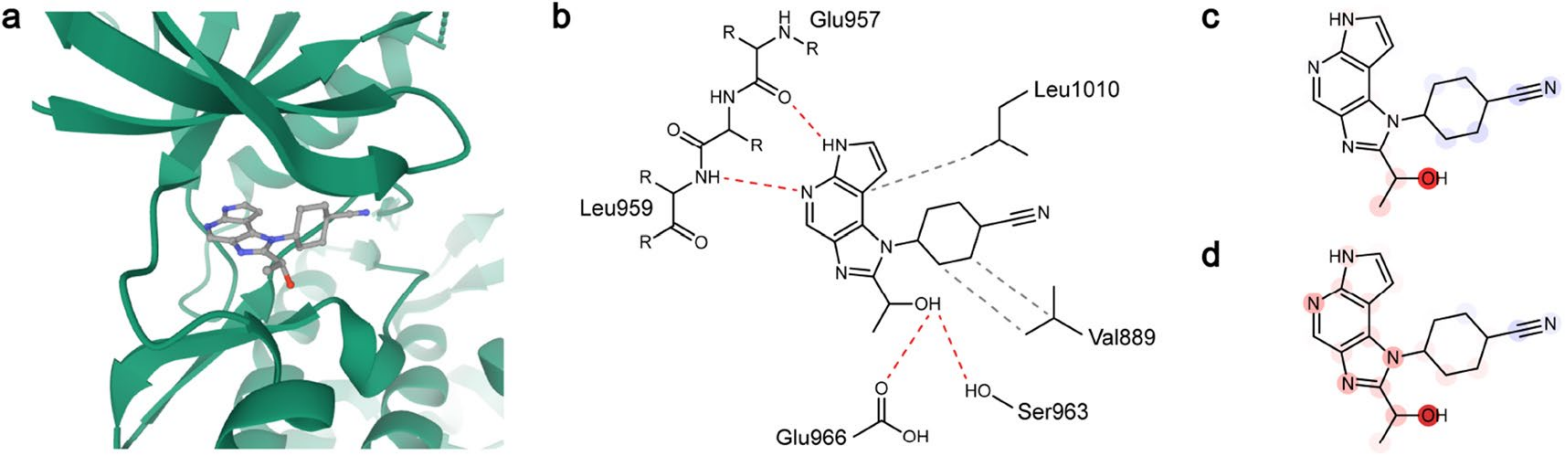
(**a**) Three-dimensional structure of the protein–ligand complex, 4IVB. The protein is depicted in a cartoon (green), and the ligand is depicted in color-coded ball-and-stick. Atom colors: gray (carbon), red (oxygen), and blue (nitrogen). (**b**) Knowledge-based estimation of protein–ligand interactions. Hydrogen bonds are depicted in red dashed lines, and hydrophobic contacts are depicted in gray dashed lines. (**c**) Heat map for the atomic contributions on the prediction of *K*_d_, obtained from the LRP on PotentialNet. (**d**) Heat map for the atomic contributions on the prediction of *K*_d_, obtained from the LRP on InteractionNet. The contributions are illustrated with color intensity of red (positive influence), white (zero influence), and blue (negative influence) colors.

The explainability techniques interpret the trained model or their predictions into explanations in human terms, which can be assessed by knowledge-based analysis. By analyzing the system with explainability techniques, the models that fail to learn appropriate knowledge to perform predictions based on valid information and fall into the “Clever Hans” decision made by fragmentary knowledge could be identified^35^. To explore the explainability of the trained InteractionNet model on the *K*_d_ prediction, we conducted the post hoc explanation on individual predictions by the LRP^32,33^. The LRP calculates the relevance for every neuron by reversely propagating, through the network, from the predicted output to the input level, and the relevance represents the quantitative contribution of a given neuron to the prediction. We used three LRP rules, LRP-0, LRP-ε, and LRP-γ, sequentially from the output layer to the input layer for production of the relevance for the neurons (Eqs. 7–9)

7 $$\mathrm{LRP}-0: {R}_{j}=\sum_{k}\frac{{a}_{j}{w}_{jk}}{{\sum }_{0,j}{a}_{j}{w}_{jk}}{R}_{k}$$

8 $$\mathrm{LRP}-\varepsilon : {R}_{j}=\sum_{k}\frac{{a}_{j}{w}_{jk}}{\varepsilon +{\sum }_{0,j}{a}_{j}{w}_{jk}}{R}_{k}$$

9 $$\mathrm{LRP}-\upgamma : {R}_{j}=\sum_{k}\frac{{a}_{j}{(w}_{jk}+\gamma {w}_{jk}^{+})}{{\sum }_{0,j}{a}_{j}{(w}_{jk}+\gamma {w}_{jk}^{+})}{R}_{k}$$

where $j$ and $k$ represent neurons at two consecutive layers, $R$ is the relevance, $a$ denotes lower layer activations, ${w}^{+}$ is a positive weight, and $\upepsilon$ and $\upgamma$ are the parameters used in each LRP rule. Once we obtained the relevance for the atomic features in the *K*_d_ prediction, it was converted to the atomic contributions by summation of relevance for individual features of the same atom. Finally, we compared the relevance with the knowledge-based analysis data from the information on hydrogen bonds and hydrophobic contacts within the complex (Fig. 4, see “Methods” for detail). Three protein–ligand complexes from the test set, PDB codes 1KAV, 3F7H, and 4IVB, were sampled and analyzed (Figs. 5–7).

#### PDB 1KAV: human tyrosine phosphatase 1B and a phosphotyrosine-mimetic inhibitor (ChEMBL1161222)

As seen in the 3D structure, half of the ligand is surrounded by the protein pocket with substantial hydrogen bonding on one of the phosphate groups. The half with the other phosphate is exposed freely to the exterior (Fig. 5a,b). On the knowledge-based protein–ligand interaction analysis, 6 hydrogen bonds were observed on the phosphate group from Ser216, Ile219, Gly220, and Arg221, and 5 hydrophobic contacts were expected on Tyr46, Phe182, and Ala217 with the aliphatic chain in the middle part of the ligand structure. ChEMBL1161222 is structurally symmetric, and it is highly important to examine whether a model properly distinguishes the two phosphate groups present in different surroundings. The heat map for the obtained atomic contributions of ChEMBL1161222 from the trained InteractionNet, arguably, showed a high correlation between human understanding and the machine-provided explanation (Fig. 5d). Notably, InteractionNet focused on only one phosphate group, which resided inside the protein pocket, predicted its high contribution to the increase in binding affinity. In comparison, the heat map of PotentialNet (Fig. 5c) considered the other phosphate group, exposed to the outside, as the one that increased the binding affinity, in addition to the one internal to the pocket. The influence of hydrophobic contacts was not observed in both heat maps of 1KAV.

#### PDB 3F7H: baculoviral IAP repeat-containing protein 7 with an azabicyclooctane-based antagonist (ChEMBL479725)

ChEMBL479725 can be divided into two parts by azabicyclooctane, the amide chain with one secondary amine, and the diphenylacetamide group. On the knowledge-based analysis, the amine and amide parts bound to 3F7H by four hydrogen bonds with their carboxyl and amide groups, and the diphenylacetamide group did not have interactions, except for one hydrophobic contact (Fig. 6a,b). InteractionNet showed a highly positive focus on the terminal amine that participated in two hydrogen bonds (Asp138 and Glu143), which was not observed in the heat map of PotentialNet (Fig. 6c,d). Compared with the strong positive relevance in InteractionNet, the LRP result of PotentialNet predicted the affinity-decreasing influence of the amine in 3F7H. A small positive focus on the azabicyclooctane ring that participated in two hydrophobic contacts with the indole (Try147) and isobutyl (Leu131) groups was observed in both models. The diphenylacetamide group was predicted to slightly decrease the *K*_d_ value, and the amide groups in the azabicyclooctane ring and the diphenylacetamide group had a negligible contribution to *K*_d_, which concurred with the knowledge-based observation.

#### PDB 4IVB: tyrosine-protein kinase JAK1 with an imidazopyrrolopyridine-based inhibitor (ChEMBL2386633)

In the 4IVB complex, ChEMBL2386633 resided in between the two lobes of JAK1 and was expected to have four hydrogen bonds, i.e., two in the imidazopyrrolopyridine group and two in the hydroxyl group and three hydrophobic contacts with JAK1 (Fig. 7a,b). The ChEMBL2386633 heat maps from both models showed a similar contribution pattern, predicting a highly positive contribution from the oxygen atom of primary alcohol, which participated in two hydrogen bonds with Ser963 and Glu966 of JAK1. All the four nitrogen atoms in imidazopyrrolopyridine were given a positive contribution in InteractionNet, although only two nitrogen atoms participated in the hydrogen bond. In comparison, the contributions of the nitrogen atoms to binding affinity were not observed in PotentialNet. The prediction on the cyanocyclohexyl group was not influential to the *K*_d_ in both models, which corresponded with the 3D structure showing the exposure of the group to the exterior.

The LRP results strongly showed the advantages of InteractionNet in two distinct points: the easily recognizable explanation powered by graph-based approach compared with image-based CNN models; the better correlation between knowledge-based and machine-generated explanation. The LRP technique could be applied similarly to the CNN models, but the relevance outcomes correspond to the voxels in the image, whereas those in the GNN models directly correspond to the nodes in the molecular graph. For generating the atomic influence on binding affinity prediction, careful separation of each voxel into atoms is necessary in the CNN models, but ambiguity in separation would be inevitable. In contrast, because the relevance in the GNN models corresponds to the node in a graph, the obtained relevance can be directly interpreted as the atomic influence. In the comparison with PotentialNet, which is another GNN model used for the prediction of protein–ligand affinity, InteractionNet showed better explanation performance on relating of the binding affinities with the hydrogen bonds between proteins and ligands. Both models exhibited good accuracy in the identification of the atoms that participated in the protein–ligand interactions, but the heat maps obtained from InteractionNet provided more precise explanations in match with the hydrogen bond patterns generated by chemical knowledge.

Through the LRP experiment and consequent visualization on the deep-learning predictions, we performed the qualitative assessment on the prediction basis of InteractionNet. The successful recognition of actual hydrogen-bond sites between proteins and ligands by InteractionNet implied two successfully learned features—recognition of hydrogen-bond-available functional groups and recognition of contact surface between the molecules. In the heat maps from the LRP experiment, the significant preference of positive contribution for the hydrogen-bond-participating heteroatoms was observed, compared with the near-zero or negative contributions of hydrocarbon chains and benzyl groups, which indicated the proper aggregation of local structures from atomic information. Distinguishing the functional groups from a given molecular graph is not only essential for efficiently modeling the protein–ligand complex formation, but also beneficial in diverse chemical tasks having both unimolecular and multimolecular characteristics. Moreover, it is indispensable for the precise prediction of binding affinity to recognize the absolute proximity of a functional group in the ligand to the active site of a protein. Our heat map analysis showed that the functional groups that existed the outside of contact surface did not increase the binding-affinity prediction in the case of protein–ligand complex having multiple hydrogen bond donor and acceptors, which indicated the InteractionNet’s recognizing ability for actual hydrogen bonds from a spatial structure.

## Conclusions

In conclusion, we presented a graph neural network (GNN) that modeled the noncovalent (NC) interactions and discussed the in-depth analysis of the model combined with the explainability technique for understanding deep-learning prediction. In the graph-based deep-learning models, there has been less attention to the NC interactions compared with the bonded interactions because of the ambiguity of NC connectivity. InteractionNet, presented herein, showed satisfactory predictive-ability for predicting the dissociation constant with RMSE of 1.321 on the PDBbind v2018 dataset. The NC convolution layers enhanced InteractionNet’s prediction accuracy, even without the utilization of the traditional bonded connectivity. We further demonstrated that InteractionNet successfully captured the important NC interactions between a protein and a ligand from a given complex through posthoc LRP analysis. The visualization of the atomic contributions showed a strong correlation with the actual hydrogen bonds in the complex. In the case of the ligand that had multiple hydrogen-bond donors and acceptors, the positive atomic contributions were observed only on the atoms participating in the actual hydrogen bonds. We believe that our model would widen the applicable tasks of the chemical, deep-learning models to the problems beyond the bonded interactions within a single molecule and also provide a meaningful explanation for the prediction, enabling the real-world applications that require prediction evidence and reliability.

## Methods

### Dataset

We employed the PDBbind v2018 dataset for the evaluation target of our InteractionNet models^24,25^. We used the refined set from the provided dataset, consisting of 4462 protein–ligand complexes with their experimentally measured *K*_d_ values. Initially, all protein–ligand data were loaded by RDKit 2019.09.2^37^ and Openbabel 3.0.0^38^, and inspected for improper conformation. During the inspection process, the molecules that failed for loading were excluded from the training dataset for further tensorization. We also excluded the complexes that had the interatomic distance below 1 Å and/or the atomic collisions in the provided 3D molecular structures. Our inspection on the sanity of the molecular structure was performed to avoid any misguided training of the model with chemically abnormal data. The noncovalent adjacency relied heavily on the distances between the atoms in the complex, and the model without the sanity check would lead to inappropriate conclusions. After inspection, 4186 protein–ligand complexes were obtained (see the Supporting Information for entire list of the PDB codes). The protein structure was cropped by retrieving the atoms of a protein within the range cutoff (3, 4, 5, or 6 Å), and the size of the protein–ligand complex structure was reduced for faster training. Only heavy atoms were considered in the entire preparation. Atomic features for building the feature matrix are listed in Table S1. For cross-validation of the model performance, we applied the 20-fold repeated random sub-sampling strategy, in which the refined set was randomly split into a training set, a validation set, and a test set on an 8:1:1 ratio in each cross-validation experiment. Twenty results were obtained through 20-fold cross-validation, and the averaged results were reported.

### Network training and evaluation

All models were implemented by using TensorFlow 2.0.0^39^ on Python 3.6.9. The training was controlled by learning-rate scheduling, early-stopping techniques, and gradient norm scaling. The learning rate was initially set to 0.00015 and lessened by a factor of 0.75 when the validation loss did not decrease within the previous 200 epochs, and the termination proceeded when the loss stopped decreasing for the previous 400 epochs. To avoid gradient exploding, a clipping parameter of 0.5 was used for gradient norm scaling. For the loss function, mean-squared-error (MSE) was used and optimized by the Adam optimizer^40^. The list of hyperparameters explored is described in Table S2. All experiments were conducted on an NVIDIA GTX 1080Ti GPU, an NVIDIA RTX 2080Ti GPU, or an NVIDIA RTX Titan GPU. Source code is publicly available at the author’s GitHub repository (https://github.com/blackmints/InteractionNet).

### Layer-wise relevance propagation (LRP)

We performed the LRP as a post-modeling explainability method. Three LRP rules, LRP-0, LRP-ε, and LRP-γ, were used for the calculation of relevance on each layer from the trained model. We adopted the LRP-0 for the output layer, LRP-ε for the FC layers, and the LRP-γ for the CV_[C]_ and CV_[NC]_ layers, based on the guideline described elsewhere^32,33^. Obtained relevance for the atomic feature was reduced to the atomic contribution by summation across features. The graph-embedding layers were omitted for the relevance calculation, because the graph-embedding layers only redistributed the relevance between features, not between atoms, resulting in the same atomic contribution before and after redistribution. For the parameters ε and γ, 0.25 was used for all LRP-ε, and 100 was used for all LRP-γ layers. The cross-validation trial that was most similar to the average in root-mean-squared-error (RMSE) was used for LRP analysis, and the LRP examples were chosen from the test set of the trial, which were predicted accurately, for comparison with knowledge-based analysis. Three-dimensional visualization of the molecular structure was obtained by Mol*^41^, and the expected hydrogen bonds and hydrophobic contacts were determined by the rules RCSB PDB use^42–45^.

## Supplementary information

Supplementary Information.
