# Supplementary material for: Layer-wise relevance propagation of InteractionNet explains protein–ligand interactions at the atom level
Source: Sci Rep. 2020 Dec 3;10:21155. doi: 10.1038/s41598-020-78169-6 (PMC7713352; doi:10.1038/s41598-020-78169-6)
Supplement: Supplementary file 1 — Supplementary Information. [file 41598_2020_78169_MOESM1_ESM.docx]

**SUPPLIMENTARY INFORMATION**

**Layer-Wise Relevance Propagation of InteractionNet Explains Protein-Ligand Interactions at the Atom Level**

**Hyeoncheol Cho**^1^, **Eok Kyun Lee**^1*^, **and Insung S. Choi**^1*^

^1^Department of Chemistry, KAIST, Daejeon 34141, Korea.

*eklee@kaist.ac.kr, ischoi@kaist.ac.kr

**CONTENTS**

• **List of the 4186 PDB codes obtained from the data refinement step.**

• **Table S1.** Atom features used in the graph representation of molecules.

• **Table S2.** Hyperparameters explored for InteractionNet.

• **Figure S1.** (Left) Scatterplots and (right) error distributions of predicted dissociation constants and experimental values included in the test set across the best, averaged, and worst cross-validation trials.

**List of PDB codes obtained from refinement step.**

10gs, 184l, 185l, 186l, 187l, 188l, 1a1e, 1a28, 1a30, 1a4k, 1a4r, 1a4w, 1a69, 1a94, 1a99, 1a9m, 1a9q, 1aaq, 1adl, 1ado, 1afk, 1afl, 1ai4, 1ai5, 1ai7, 1aid, 1aj7, 1ajn, 1ajp, 1ajq, 1ajv, 1ajx, 1alw, 1amk, 1amw, 1apv, 1atl, 1atr, 1avn, 1ax0, 1azm, 1b05, 1b0h, 1b1h, 1b2h, 1b32, 1b38, 1b3f, 1b3g, 1b3h, 1b3l, 1b40, 1b46, 1b4h, 1b4z, 1b51, 1b52, 1b55, 1b57, 1b58, 1b5h, 1b5i, 1b6h, 1b6j, 1b6k, 1b6l, 1b7h, 1b8n, 1b8o, 1b8y, 1b9j, 1bai, 1bcd, 1bcu, 1bdq, 1bgq, 1bhf, 1bhx, 1bju, 1bjv, 1bm7, 1bma, 1bn1, 1bn3, 1bn4, 1bnn, 1bnq, 1bnt, 1bnu, 1bnv, 1bnw, 1bp0, 1bq4, 1br6, 1bty, 1bv7, 1bv9, 1bwa, 1bwb, 1bxo, 1bxq, 1bxr, 1bzc, 1bzj, 1bzy, 1c1r, 1c1u, 1c1v, 1c3x, 1c4u, 1c5c, 1c5n, 1c5o, 1c5p, 1c5q, 1c5s, 1c5t, 1c5x, 1c5y, 1c70, 1c83, 1c84, 1c86, 1c87, 1c88, 1cbx, 1ceb, 1cet, 1ciz, 1cnw, 1cnx, 1cny, 1ctt, 1d09, 1d2e, 1d3d, 1d3p, 1d4h, 1d4i, 1d4j, 1d4k, 1d4l, 1d4p, 1d4y, 1d6v, 1d6w, 1d7i, 1d7j, 1d9i, 1dar, 1det, 1df8, 1dgm, 1dhi, 1dhj, 1dif, 1dl7, 1dmp, 1dqn, 1drj, 1drk, 1drv, 1dud, 1duv, 1dy4, 1dzk, 1e1v, 1e1x, 1e2k, 1e2l, 1e3g, 1e3v, 1e4h, 1e5j, 1e66, 1e6s, 1eb2, 1ebw, 1eby, 1ebz, 1ec0, 1ec1, 1ec2, 1ec3, 1ec9, 1ecq, 1ecv, 1efy, 1egh, 1ejn, 1ela, 1elb, 1elc, 1eld, 1ele, 1elr, 1enu, 1eoc, 1epo, 1erb, 1ex8, 1ez9, 1ezq, 1f0r, 1f0s, 1f0t, 1f0u, 1f3e, 1f4e, 1f4f, 1f4g, 1f4x, 1f5k, 1f5l, 1f73, 1f74, 1f8b, 1f8c, 1f8d, 1f8e, 1fao, 1fch, 1fcx, 1fcy, 1fcz, 1fd0, 1fh7, 1fh8, 1fh9, 1fhd, 1fiv, 1fjs, 1fkb, 1fkf, 1fkg, 1fki, 1fkn, 1fl3, 1flr, 1fm9, 1fo0, 1fpc, 1fq5, 1ftm, 1fv0, 1fzj, 1fzk, 1fzm, 1fzo, 1fzq, 1g1d, 1g2k, 1g2l, 1g2o, 1g30, 1g32, 1g35, 1g36, 1g3d, 1g3e, 1g45, 1g48, 1g4o, 1g52, 1g53, 1g54, 1g74, 1g7f, 1g7g, 1g7q, 1g85, 1g98, 1gaf, 1gai, 1gar, 1gfy, 1ghv, 1ghw, 1ghy, 1ghz, 1gi1, 1gi4, 1gi7, 1gj6, 1gjc, 1gnm, 1gnn, 1gno, 1gpk, 1gpn, 1grp, 1gvw, 1gvx, 1gwv, 1gx8, 1gyx, 1gyy, 1h0a, 1h1s, 1h22, 1h23, 1h2k, 1h2t, 1h46, 1h4w, 1h5v, 1h6h, 1hbv, 1hi3, 1hi4, 1hi5, 1hih, 1hii, 1hk4, 1hlk, 1hmr, 1hms, 1hmt, 1hn4, 1hos, 1hp5, 1hpo, 1hps, 1hpv, 1hpx, 1hsh, 1hsl, 1hvh, 1hvi, 1hvj, 1hvk, 1hvl, 1hvr, 1hvs, 1hwr, 1hxb, 1hxw, 1hyo, 1i1e, 1i2s, 1i37, 1i5r, 1i7z, 1i9n, 1i9p, 1ie9, 1if7, 1if8, 1igj, 1ii5, 1iih, 1iiq, 1ik4, 1ikt, 1ivp, 1izh, 1izi, 1j01, 1j14, 1j16, 1j17, 1j36, 1j37, 1j4r, 1jak, 1jao, 1jaq, 1jcx, 1jet, 1jeu, 1jev, 1jgl, 1jlr, 1jmf, 1jmg, 1jn4, 1jq8, 1jqy, 1jsv, 1jvu, 1jyq, 1jys, 1jzs, 1k1i, 1k1j, 1k1l, 1k1m, 1k1n, 1k1o, 1k1y, 1k21, 1k22, 1k27, 1k4g, 1k4h, 1k6c, 1k6p, 1k6t, 1k6v, 1k9s, 1kav, 1kc7, 1kdk, 1kel, 1kjr, 1km3, 1kmy, 1koj, 1kpm, 1ksn, 1kug, 1kui, 1kuk, 1kv1, 1kv5, 1kyv, 1kzk, 1kzn, 1l83, 1l8g, 1laf, 1lag, 1lah, 1lbf, 1lbk, 1lee, 1lf2, 1lgt, 1lgw, 1lhu, 1li2, 1li3, 1li6, 1lke, 1lkk, 1lkl, 1lnm, 1loq, 1lpg, 1lpk, 1lpz, 1lst, 1lvu, 1lyb, 1lyx, 1lzq, 1m0b, 1m0n, 1m0o, 1m0q, 1m1b, 1m2p, 1m2q, 1m2r, 1m2x, 1m48, 1m4h, 1m5w, 1m7d, 1m7i, 1m7y, 1m83, 1mai, 1mes, 1met, 1mfa, 1mfd, 1mfi, 1mh5, 1mjj, 1mmq, 1mmr, 1moq, 1mq5, 1mq6, 1mrn, 1mrs, 1mrw, 1mrx, 1msm, 1msn, 1mtr, 1mu6, 1mu8, 1mue, 1my4, 1n0s, 1n1m, 1n3i, 1n46, 1n4h, 1n4k, 1n5r, 1nc1, 1nc3, 1ndv, 1nf8, 1nfu, 1nfw, 1nfx, 1nfy, 1nh0, 1nhu, 1nhz, 1nja, 1njc, 1njd, 1nje, 1njs, 1nki, 1nl9, 1nli, 1nm6, 1nny, 1no6, 1np0, 1nq7, 1nt1, 1nvq, 1nvr, 1nvs, 1nw4, 1nw5, 1nw7, 1nwl, 1nz7, 1o0f, 1o0h, 1o0m, 1o0n, 1o1s, 1o2h, 1o2j, 1o2n, 1o2o, 1o2q, 1o2r, 1o2w, 1o2z, 1o30, 1o33, 1o35, 1o38, 1o3d, 1o3f, 1o3i, 1o3j, 1o3l, 1o5a, 1o5c, 1o5e, 1o5g, 1o7o, 1o86, 1oar, 1oau, 1oba, 1ocq, 1odi, 1odj, 1oe8, 1ogd, 1ogg, 1ogx, 1ogz, 1ohr, 1oif, 1okl, 1om1, 1ony, 1onz, 1ork, 1os0, 1os5, 1oss, 1owe, 1owh, 1oxr, 1oyq, 1oyt, 1oz0, 1p19, 1p1n, 1p1o, 1p1q, 1p57, 1pa9, 1pb8, 1pb9, 1pbq, 1pdz, 1pfu, 1pgp, 1phw, 1pkx, 1pme, 1pot, 1ppc, 1pph, 1ppi, 1ppk, 1ppl, 1ppm, 1pr5, 1pro, 1pvn, 1px4, 1pxn, 1pxo, 1pxp, 1pyn, 1pz5, 1pzi, 1pzp, 1q1g, 1q54, 1q5k, 1q65, 1q72, 1q7a, 1q84, 1q8t, 1q8u, 1q8w, 1q91, 1qan, 1qaw, 1qb1, 1qb6, 1qb9, 1qbn, 1qbo, 1qbq, 1qbr, 1qbs, 1qbt, 1qbu, 1qbv, 1qf0, 1qf1, 1qf2, 1qft, 1qhc, 1qin, 1qji, 1qk3, 1qk4, 1qka, 1qkb, 1qkt, 1ql7, 1ql9, 1qxk, 1qy1, 1qy2, 1qyg, 1r0p, 1r5y, 1r9l, 1rbp, 1rd4, 1rjk, 1rmz, 1rnm, 1rnt, 1rp7, 1rpf, 1rpj, 1rql, 1rr6, 1rtf, 1s19, 1s38, 1s39, 1s5z, 1s89, 1sb1, 1sbg, 1sdt, 1sdu, 1sdv, 1sgu, 1sh9, 1siv, 1sl3, 1sld, 1sln, 1sqa, 1sqo, 1sqt, 1sr7, 1srg, 1ssq, 1stc, 1str, 1sv3, 1sw2, 1swg, 1swr, 1syh, 1syi, 1szd, 1t31, 1t32, 1t4v, 1t7d, 1t7j, 1ta6, 1tcx, 1td7, 1thz, 1tjp, 1tmn, 1tng, 1tnh, 1tni, 1tom, 1tpw, 1tq4, 1trd, 1tsy, 1ttm, 1tx7, 1u0g, 1u1b, 1u1w, 1u33, 1u71, 1ua4, 1ucn, 1ugx, 1ui0, 1uj5, 1uou, 1upf, 1ur9, 1usi, 1usk, 1utj, 1utl, 1utm, 1utn, 1uto, 1uv6, 1uvt, 1uw6, 1uwf, 1uwt, 1uwu, 1uz1, 1uz4, 1uz8, 1v0l, 1v2j, 1v2k, 1v2l, 1v2n, 1v2o, 1v2r, 1v2s, 1v2t, 1v2u, 1v2w, 1v48, 1vfn, 1vso, 1vyf, 1vyg, 1vzq, 1w0z, 1w11, 1w13, 1w3j, 1w3k, 1w4o, 1w4p, 1w4q, 1w5v, 1w5w, 1w5x, 1w5y, 1w7g, 1w96, 1w9u, 1w9v, 1wc1, 1wcq, 1wdn, 1wht, 1wm1, 1wn6, 1ws1, 1ws4, 1wur, 1wvj, 1x1z, 1x38, 1x39, 1x8d, 1x8j, 1x8r, 1x8t, 1xap, 1xbo, 1xd0, 1xff, 1xgi, 1xh4, 1xh5, 1xh9, 1xhy, 1xjd, 1xk9, 1xka, 1xkk, 1xow, 1xpz, 1xq0, 1xr9, 1xt8, 1xug, 1xws, 1y0l, 1y1z, 1y20, 1y3n, 1y3p, 1y3v, 1y3x, 1y6q, 1y6r, 1yc1, 1yc4, 1yda, 1ydb, 1ydd, 1ydk, 1ydr, 1yds, 1ydt, 1yei, 1yej, 1yet, 1yfz, 1yp9, 1ype, 1ypg, 1ypj, 1yq7, 1yqj, 1yqy, 1z1h, 1z4o, 1z6e, 1z6s, 1z71, 1z95, 1z9g, 1z9y, 1zc9, 1zdp, 1zea, 1zfq, 1zge, 1zgi, 1zhy, 1zoe, 1zog, 1zoh, 1zp8, 1zpa, 1zs0, 1zsf, 1zvx, 2a14, 2a4m, 2a5c, 2a5s, 2a8g, 2aac, 2afw, 2afx, 2aj8, 2al5, 2am4, 2amt, 2ans, 2aoc, 2aod, 2aoe, 2aqu, 2arm, 2avm, 2avo, 2avs, 2ax9, 2ayr, 2azr, 2b07, 2b1g, 2b1i, 2b4l, 2b7d, 2b9a, 2baj, 2bak, 2bal, 2bes, 2bet, 2bfq, 2bfr, 2bmk, 2bo4, 2boh, 2boj, 2bok, 2bpv, 2bpy, 2bq7, 2bqv, 2br1, 2brb, 2brm, 2bt9, 2buv, 2bvd, 2bvr, 2bvs, 2byr, 2bys, 2bz6, 2bza, 2c1p, 2c3i, 2c3l, 2c80, 2c92, 2c94, 2c97, 2ca8, 2cbj, 2cbu, 2cbv, 2cbz, 2cc7, 2ccb, 2ccc, 2ce9, 2cej, 2cen, 2ceq, 2cer, 2ces, 2cet, 2cex, 2cf8, 2cf9, 2cgf, 2cgr, 2cht, 2cle, 2clh, 2cli, 2clk, 2cn0, 2csn, 2d0k, 2d1n, 2d1o, 2d3u, 2d3z, 2doo, 2drc, 2dri, 2dw7, 2e1w, 2e27, 2e2p, 2e2r, 2e7f, 2e91, 2e92, 2e94, 2e9u, 2epn, 2erz, 2euk, 2evl, 2ewa, 2ews, 2exm, 2ez7, 2f1g, 2f2h, 2f34, 2f35, 2f6t, 2f7i, 2f81, 2f8g, 2f94, 2f9k, 2fdp, 2fgu, 2fgv, 2fle, 2flr, 2fmb, 2fpz, 2fqo, 2fqt, 2fqw, 2fqx, 2fqy, 2fu8, 2fvd, 2fw6, 2fx6, 2fxs, 2fxu, 2fxv, 2fzc, 2fzg, 2fzk, 2g5u, 2g94, 2gh9, 2gj5, 2gkl, 2gl0, 2glp, 2gss, 2gst, 2gv6, 2gv7, 2gvj, 2gvv, 2gyi, 2gz2, 2gzl, 2h15, 2h21, 2h3e, 2h4g, 2h4k, 2h4n, 2h6b, 2h6t, 2ha2, 2ha3, 2ha6, 2hah, 2haw, 2hb1, 2hb3, 2hhn, 2hjb, 2hkf, 2hl4, 2hmu, 2hmv, 2hnc, 2hnx, 2hoc, 2hu6, 2hxm, 2hzl, 2i0a, 2i19, 2i2c, 2i3h, 2i3i, 2i4d, 2i4j, 2i4u, 2i4v, 2i4w, 2i4x, 2i4z, 2i6b, 2i80, 2idw, 2ihj, 2ihq, 2iko, 2isw, 2iuz, 2iwx, 2izl, 2j27, 2j2u, 2j34, 2j47, 2j4g, 2j4i, 2j62, 2j75, 2j77, 2j78, 2j79, 2j7b, 2j7d, 2j7e, 2j7f, 2j7g, 2j7h, 2j94, 2j95, 2jdm, 2jdp, 2jds, 2jdu, 2jf4, 2jfz, 2jg0, 2jgs, 2jh0, 2jh5, 2jh6, 2jiw, 2jjb, 2jke, 2jkh, 2jkp, 2jxr, 2mas, 2nmx, 2nmz, 2nn1, 2nn7, 2nnd, 2nsj, 2nsl, 2nt7, 2nta, 2o0u, 2o4j, 2o4k, 2o4l, 2o4n, 2o4p, 2o4r, 2o4s, 2o4z, 2o8h, 2oag, 2oax, 2oc2, 2ogy, 2oi0, 2oi2, 2oiq, 2ojg, 2ojj, 2olb, 2ole, 2on6, 2ot1, 2ovv, 2ovy, 2oxd, 2oxn, 2oxx, 2oxy, 2oym, 2p09, 2p15, 2p16, 2p2a, 2p3a, 2p3b, 2p3c, 2p3i, 2p4j, 2p4s, 2p4y, 2p53, 2p7a, 2p7g, 2p7z, 2p95, 2pbw, 2pcp, 2pgz, 2pk5, 2pk6, 2pog, 2pou, 2pov, 2pow, 2pq9, 2pqb, 2pqc, 2pql, 2pqz, 2psu, 2psv, 2ptz, 2pu1, 2pu2, 2pv1, 2pvh, 2pvj, 2pvk, 2pvl, 2pvm, 2pvu, 2pwc, 2pwd, 2pwg, 2pwr, 2py4, 2pyn, 2pyy, 2q1q, 2q2a, 2q38, 2q54, 2q55, 2q5k, 2q63, 2q64, 2q6f, 2q7q, 2q88, 2q89, 2q8h, 2q8m, 2q8z, 2qbp, 2qbq, 2qbr, 2qbs, 2qbu, 2qbw, 2qci, 2qd6, 2qd7, 2qd8, 2qdt, 2qe4, 2qg0, 2qg2, 2qhy, 2qhz, 2qi0, 2qi1, 2qi3, 2qi4, 2qi5, 2qi6, 2qi7, 2qm9, 2qmg, 2qnn, 2qnp, 2qnq, 2qpq, 2qpu, 2qrk, 2qrl, 2qtg, 2qtn, 2qtt, 2qu6, 2qw1, 2qwb, 2qwc, 2qwd, 2qwe, 2qwf, 2qzr, 2r0h, 2r0z, 2r1y, 2r23, 2r2m, 2r2w, 2r38, 2r3t, 2r3w, 2r43, 2r58, 2r59, 2r5a, 2r5p, 2r75, 2r9w, 2r9x, 2ra0, 2ra6, 2rcb, 2rcn, 2rd6, 2reg, 2ri9, 2rin, 2rio, 2rk8, 2rkd, 2rke, 2rkf, 2rkg, 2sim, 2std, 2tmn, 2tpi, 2uwd, 2uwl, 2uwo, 2uwp, 2uxi, 2uxz, 2uy0, 2uy3, 2uy4, 2uy5, 2uyn, 2uyq, 2v00, 2v25, 2v2c, 2v2h, 2v2q, 2v2v, 2v3d, 2v3u, 2v54, 2v57, 2v58, 2v59, 2v77, 2v7a, 2v88, 2v95, 2vb8, 2vba, 2vc9, 2ves, 2vfk, 2vh0, 2vh6, 2vhj, 2vjx, 2vk2, 2vk6, 2vkm, 2vl4, 2vmc, 2vmd, 2vmf, 2vnp, 2vnt, 2vo4, 2vo5, 2vpe, 2vpn, 2vpo, 2vqt, 2vrj, 2vsl, 2vt3, 2vuk, 2vvc, 2vvn, 2vvs, 2vvu, 2vvv, 2vw1, 2vw2, 2vw5, 2vwc, 2vwl, 2vwn, 2vwo, 2vxn, 2vyt, 2vzr, 2w08, 2w26, 2w47, 2w4x, 2w5g, 2w66, 2w67, 2w8j, 2w8w, 2w8y, 2w9h, 2wb5, 2wbg, 2wc3, 2wc4, 2wca, 2we3, 2web, 2wec, 2wed, 2weg, 2weh, 2wej, 2weo, 2weq, 2wer, 2wf5, 2wgj, 2whp, 2wjg, 2wk6, 2wky, 2wkz, 2wl0, 2wly, 2wlz, 2wm0, 2wn9, 2wnc, 2wnj, 2wor, 2wos, 2wq5, 2wr8, 2wtv, 2wuf, 2wvt, 2wvz, 2wyf, 2wyg, 2wyj, 2wzf, 2wzm, 2wzs, 2x00, 2x09, 2x0y, 2x2r, 2x4z, 2x6x, 2x7t, 2x7u, 2x8z, 2x91, 2x95, 2x96, 2x97, 2xab, 2xb7, 2xb8, 2xbp, 2xbv, 2xbw, 2xbx, 2xc0, 2xc4, 2xd9, 2xda, 2xde, 2xdk, 2xdl, 2xdx, 2xg9, 2xhm, 2xht, 2xib, 2xii, 2xj1, 2xj2, 2xj7, 2xjg, 2xjj, 2xjx, 2xm1, 2xm2, 2xmy, 2xn3, 2xn5, 2xnb, 2xog, 2xp7, 2xpk, 2xxr, 2xxt, 2xxx, 2xyd, 2xye, 2xyf, 2xys, 2xyt, 2y5f, 2y5g, 2y5h, 2y7i, 2y7x, 2y7z, 2y80, 2y81, 2y82, 2y8c, 2ya6, 2ya7, 2ya8, 2yay, 2yaz, 2yb0, 2ydt, 2ydw, 2yek, 2yel, 2yfa, 2yfe, 2yfx, 2yge, 2ygf, 2yi0, 2yi7, 2yix, 2yk1, 2yki, 2ylc, 2ymd, 2yme, 2ypi, 2ypo, 2yxj, 2yz3, 2z1w, 2z4o, 2z94, 2za5, 2zb1, 2zc9, 2zcq, 2zcr, 2zcs, 2zda, 2zdk, 2zdl, 2zdm, 2zdn, 2zfp, 2zfs, 2zft, 2zgx, 2zkj, 2zmm, 2zn7, 2zq0, 2zq2, 2zwz, 2zx6, 2zx7, 2zx8, 2zxd, 2zxg, 2zy1, 2zym, 2zz1, 2zz2, 3a1c, 3a1d, 3a1e, 3a2o, 3a5y, 3a6t, 3a9i, 3aaq, 3aas, 3aau, 3acw, 3acx, 3ag9, 3agl, 3aid, 3alt, 3ao2, 3ao4, 3ao5, 3ap4, 3aqt, 3arp, 3arq, 3arw, 3arx, 3axz, 3b1m, 3b24, 3b25, 3b26, 3b27, 3b2q, 3b3x, 3b4f, 3b4p, 3b50, 3b5r, 3b65, 3b66, 3b67, 3b68, 3b7j, 3b7r, 3b92, 3bbb, 3bbf, 3be9, 3bex, 3bft, 3bfu, 3bgb, 3bgc, 3bgq, 3bgs, 3bgz, 3bkk, 3bkl, 3bl0, 3bl1, 3bpc, 3bqc, 3bra, 3brn, 3bu1, 3buf, 3bug, 3buh, 3bv9, 3bva, 3bvb, 3bwj, 3bxe, 3bxf, 3bxg, 3bxh, 3bzf, 3c2f, 3c2o, 3c2r, 3c2u, 3c39, 3c4h, 3c52, 3c56, 3c79, 3c84, 3cct, 3ccw, 3ccz, 3cd0, 3cd5, 3cd7, 3cda, 3cdb, 3cf8, 3cfn, 3cft, 3cj2, 3cj4, 3cj5, 3ckb, 3cke, 3ckp, 3ckz, 3cl0, 3cm2, 3cow, 3coy, 3coz, 3cs7, 3ctt, 3cyw, 3cyx, 3cyz, 3cz1, 3czv, 3d0b, 3d0e, 3d1x, 3d1y, 3d1z, 3d2e, 3d6o, 3d6p, 3d78, 3d7k, 3d7z, 3d83, 3d8w, 3d8z, 3d91, 3d9z, 3da9, 3dbu, 3dc3, 3dcc, 3dd0, 3dd8, 3dgo, 3djk, 3djo, 3djp, 3djq, 3djv, 3djx, 3dk1, 3dln, 3dnd, 3dne, 3dp4, 3dp9, 3drf, 3drg, 3dri, 3dsz, 3dyo, 3dzt, 3e3c, 3e5a, 3e5u, 3e6y, 3e92, 3e93, 3eax, 3eb1, 3ebh, 3ebi, 3ebl, 3ebo, 3ebp, 3ed0, 3eeb, 3eft, 3egt, 3ehx, 3ehy, 3eko, 3ekp, 3ekr, 3ekt, 3ekv, 3ekw, 3ekx, 3el1, 3el4, 3el5, 3el9, 3elc, 3eqr, 3ery, 3evd, 3ewj, 3exe, 3f15, 3f16, 3f17, 3f18, 3f19, 3f1a, 3f33, 3f34, 3f37, 3f3c, 3f3d, 3f3e, 3f48, 3f5j, 3f5k, 3f5l, 3f68, 3f6g, 3f70, 3f78, 3f7g, 3f7h, 3f7i, 3f8c, 3f8e, 3f8f, 3fas, 3fat, 3fcq, 3ffg, 3ffp, 3fhb, 3fj7, 3fjg, 3fl5, 3fqe, 3fql, 3fuc, 3fur, 3fuz, 3fv1, 3fv2, 3fv3, 3fvh, 3fvk, 3fvn, 3fwv, 3fzn, 3fzy, 3g0e, 3g0i, 3g0w, 3g19, 3g1d, 3g1v, 3g2y, 3g2z, 3g31, 3g32, 3g34, 3g35, 3g3r, 3g5k, 3ga5, 3gba, 3gbb, 3gbe, 3gc4, 3gc5, 3gcp, 3gcs, 3gcu, 3gdt, 3ge7, 3ggu, 3gi4, 3gi5, 3gi6, 3gjw, 3gk1, 3gkz, 3gm0, 3gnw, 3gqz, 3gr2, 3gs6, 3gsm, 3gss, 3gst, 3gt9, 3gta, 3gtc, 3gv9, 3gvb, 3gvu, 3gx0, 3gy2, 3gy3, 3gy4, 3gy7, 3h1x, 3h30, 3h5b, 3h78, 3h89, 3h8b, 3hb4, 3hcm, 3hek, 3hig, 3hit, 3hk1, 3hkn, 3hkq, 3hkt, 3hku, 3hkw, 3hky, 3hl5, 3hl7, 3hl8, 3hll, 3hmo, 3hmp, 3hp9, 3hs4, 3hu3, 3hub, 3huc, 3hv8, 3hvj, 3hzk, 3hzm, 3hzv, 3i25, 3i3b, 3i4b, 3i4y, 3i51, 3i5z, 3i60, 3i6o, 3i73, 3i7e, 3i9g, 3iae, 3ibi, 3ibl, 3ibn, 3ibu, 3ies, 3ifl, 3igp, 3ijh, 3ikd, 3ikg, 3imc, 3ime, 3iob, 3ioc, 3iod, 3ioe, 3iof, 3iog, 3ip5, 3ip6, 3ip8, 3ip9, 3iph, 3ipq, 3ipu, 3iqu, 3isj, 3iss, 3iub, 3iue, 3ivc, 3ivg, 3ivx, 3iw5, 3iw6, 3jdw, 3jrs, 3jrx, 3juk, 3juo, 3jup, 3jvr, 3jvs, 3jy0, 3jya, 3jyr, 3jzh, 3jzj, 3k00, 3k02, 3k1j, 3k2f, 3k37, 3k4d, 3k4q, 3k5v, 3k5x, 3k8c, 3k8o, 3k8q, 3k97, 3k99, 3kdb, 3kdc, 3kdd, 3kdm, 3kek, 3kgp, 3kgt, 3kgu, 3kiv, 3kjd, 3kku, 3kmc, 3kmx, 3kmy, 3kqr, 3kr8, 3kwa, 3kyq, 3l0v, 3l3l, 3l3m, 3l3n, 3l4u, 3l4v, 3l4w, 3l4x, 3l4y, 3l4z, 3l59, 3ldp, 3ldq, 3le9, 3lea, 3lir, 3liw, 3ljg, 3ljo, 3ljz, 3lk8, 3lka, 3lmk, 3lpi, 3lpk, 3lpl, 3lpp, 3lvw, 3lxe, 3lxk, 3lzs, 3lzu, 3lzz, 3m1k, 3m35, 3m36, 3m37, 3m3c, 3m3x, 3m3z, 3m40, 3m5e, 3m67, 3m6r, 3m8u, 3m96, 3mam, 3mdz, 3mf5, 3mhc, 3mhi, 3mhl, 3mhm, 3mho, 3mhw, 3mi2, 3mi3, 3miy, 3ml2, 3ml5, 3mmf, 3mna, 3mof, 3ms9, 3mss, 3muz, 3mv0, 3mxd, 3mxe, 3myg, 3myq, 3mzc, 3n0n, 3n1c, 3n2p, 3n2u, 3n2v, 3n35, 3n3g, 3n3j, 3n4b, 3n76, 3n7a, 3n7o, 3n86, 3n8k, 3n9r, 3n9s, 3nb5, 3nee, 3neo, 3nes, 3nex, 3ng4, 3nhi, 3nht, 3ni5, 3nik, 3nim, 3nkk, 3nox, 3npc, 3nq3, 3nq9, 3nsn, 3nu4, 3nu5, 3nu6, 3nu9, 3nuj, 3nuo, 3nw3, 3nx7, 3nxq, 3nyd, 3nyx, 3o4k, 3o5n, 3o5x, 3o75, 3o84, 3o8p, 3o99, 3o9a, 3o9d, 3o9e, 3o9i, 3o9p, 3oaf, 3ocp, 3ocz, 3oe4, 3oe5, 3ohi, 3oil, 3oim, 3oku, 3old, 3ouj, 3ov1, 3ove, 3ovn, 3owj, 3own, 3oy0, 3oy8, 3oyq, 3oyw, 3ozg, 3ozj, 3ozp, 3ozr, 3ozs, 3ozt, 3p17, 3p2e, 3p3r, 3p3s, 3p3t, 3p4v, 3p58, 3p5l, 3p5o, 3p7i, 3p8n, 3p8o, 3p8p, 3p8z, 3p9l, 3p9m, 3pb7, 3pb8, 3pb9, 3pbb, 3pcj, 3pck, 3pcn, 3pd8, 3pd9, 3pe1, 3pe2, 3pfp, 3pgl, 3pgu, 3pju, 3pn1, 3pn4, 3po6, 3ppm, 3ppp, 3ppq, 3ppr, 3prs, 3pwd, 3pwk, 3pwm, 3pww, 3pyy, 3q1x, 3q2j, 3q44, 3q6w, 3q6z, 3q71, 3q7q, 3qaa, 3qbc, 3qdd, 3qfd, 3qfy, 3qfz, 3qgw, 3qgy, 3qkd, 3qlm, 3qox, 3qps, 3qqa, 3qqs, 3qt6, 3qto, 3qtv, 3qwc, 3qx5, 3qx9, 3qxt, 3qxv, 3r16, 3r17, 3r1v, 3r24, 3r4m, 3r4n, 3r4p, 3r5t, 3r6u, 3r7o, 3r88, 3rdo, 3rdq, 3re4, 3rf4, 3rf5, 3rlp, 3rlq, 3rlr, 3rm4, 3rm9, 3roc, 3rr4, 3rsx, 3rt8, 3rtf, 3ru1, 3rux, 3rv4, 3rv8, 3rwp, 3ryj, 3ryv, 3ryx, 3ryy, 3ryz, 3rz0, 3rz1, 3rz5, 3rz7, 3rz8, 3s0b, 3s0d, 3s0e, 3s2v, 3s43, 3s45, 3s54, 3s5y, 3s6t, 3s71, 3s72, 3s73, 3s75, 3s76, 3s77, 3s78, 3s8l, 3s8n, 3s8o, 3s9e, 3sfg, 3sha, 3shc, 3si3, 3si4, 3sio, 3sk2, 3slz, 3sm2, 3spf, 3sr4, 3std, 3str, 3su0, 3su1, 3su2, 3su3, 3su4, 3su5, 3su6, 3sue, 3suf, 3sug, 3sur, 3sus, 3sut, 3suu, 3suv, 3suw, 3sv2, 3sw8, 3sww, 3sxf, 3t1a, 3t1m, 3t2w, 3t3c, 3t3u, 3t5u, 3t60, 3t64, 3t6b, 3t70, 3t82, 3t83, 3t84, 3t85, 3t8v, 3ta0, 3ta1, 3tao, 3tay, 3tb6, 3tcg, 3td4, 3tf6, 3tfn, 3tfp, 3tfu, 3th9, 3tif, 3tk2, 3tkw, 3tmk, 3ts4, 3tsk, 3tt4, 3ttm, 3ttp, 3tu7, 3tvc, 3twp, 3tz0, 3tza, 3tzm, 3u10, 3u5j, 3u5l, 3u6i, 3u81, 3u8j, 3u8k, 3u8l, 3u8n, 3u90, 3u92, 3u93, 3u9q, 3ubd, 3ucj, 3udd, 3udh, 3ueu, 3uev, 3uew, 3uex, 3ug2, 3ui7, 3uil, 3uj9, 3ujc, 3ujd, 3umq, 3uo4, 3uod, 3up2, 3upk, 3upv, 3uri, 3usx, 3utu, 3uu1, 3uug, 3uuo, 3uw4, 3uw5, 3uxd, 3uxk, 3uyr, 3uz5, 3uzj, 3v2n, 3v2p, 3v2q, 3v3q, 3v4t, 3v51, 3v5p, 3v5t, 3v78, 3v7x, 3vbd, 3veh, 3vf7, 3vfa, 3vfb, 3vha, 3vhc, 3vhd, 3vhk, 3vjc, 3vje, 3vtr, 3vvy, 3vw1, 3vw2, 3vx3, 3w07, 3w37, 3w5n, 3w9k, 3w9r, 3wgg, 3wha, 3wjw, 3wmc, 3wtj, 3wtl, 3wtm, 3wtn, 3wto, 3wvm, 3wz6, 3wz7, 3wz8, 3wzn, 3x00, 3zbx, 3zc5, 3zcl, 3zdg, 3zdh, 3zdv, 3zi0, 3zi8, 3zj6, 3zk6, 3zll, 3zln, 3zlr, 3zm9, 3zns, 3zps, 3zpu, 3zq9, 3zqe, 3zso, 3zsq, 3zsx, 3zsy, 3zt2, 3zt3, 3zv7, 3zxz, 3zyf, 3zyu, 3zze, 456c, 4a4q, 4a4v, 4a4w, 4a6b, 4a6c, 4a6l, 4a6s, 4a7i, 4a95, 4ab9, 4aba, 4abb, 4abe, 4abf, 4abg, 4abh, 4acc, 4aci, 4ad2, 4ad3, 4ad6, 4afg, 4ag8, 4agc, 4agl, 4agm, 4agn, 4ago, 4agp, 4agq, 4ahr, 4ahs, 4ahu, 4ai5, 4aia, 4aj4, 4aje, 4aji, 4ajl, 4alx, 4aoi, 4ap7, 4app, 4aq4, 4aq6, 4aqh, 4ara, 4arb, 4arw, 4asd, 4ase, 4asj, 4att, 4auj, 4av4, 4av5, 4avh, 4avi, 4avj, 4avs, 4ax9, 4axd, 4ayp, 4ayq, 4ayu, 4az5, 4az6, 4azb, 4azc, 4azg, 4azi, 4b0b, 4b1j, 4b2i, 4b2l, 4b32, 4b33, 4b34, 4b35, 4b3b, 4b3c, 4b3d, 4b5d, 4b5w, 4b6o, 4b6p, 4b6r, 4b6s, 4b73, 4b74, 4b76, 4b7j, 4b7p, 4b7r, 4b8y, 4b9k, 4b9z, 4bah, 4bak, 4bam, 4ban, 4bao, 4baq, 4bb9, 4bc5, 4bck, 4bcm, 4bcn, 4bco, 4bcp, 4bcs, 4bf1, 4bf6, 4bi6, 4bi7, 4bj8, 4bks, 4bkt, 4bny, 4bqg, 4bqh, 4bqs, 4bs0, 4bt3, 4bt4, 4bt5, 4btk, 4buq, 4c1t, 4c1u, 4c1y, 4c2v, 4c52, 4c5d, 4c6u, 4c9x, 4ca5, 4ca6, 4ca7, 4ca8, 4cc5, 4cd0, 4cd4, 4cd5, 4ceb, 4cfl, 4cgi, 4cig, 4ciw, 4cj4, 4cjp, 4cjq, 4cjr, 4ck3, 4cl6, 4clj, 4cmo, 4cp5, 4cp7, 4cpr, 4cps, 4cpt, 4cpy, 4cpz, 4cr5, 4cr9, 4cra, 4crb, 4crc, 4crf, 4crl, 4csd, 4css, 4cst, 4cu7, 4cu8, 4cwf, 4cwn, 4cwo, 4cwp, 4cwq, 4cwr, 4cws, 4cwt, 4czs, 4d1j, 4d3h, 4d4d, 4d7b, 4d8z, 4da5, 4daf, 4db7, 4dbm, 4dcs, 4ddh, 4ddk, 4ddm, 4de0, 4de1, 4de2, 4de5, 4del, 4der, 4det, 4deu, 4dff, 4dfg, 4djo, 4djp, 4djq, 4djr, 4dju, 4djv, 4djw, 4djx, 4djy, 4dko, 4dkp, 4dkq, 4dkr, 4dld, 4dmw, 4do4, 4do5, 4dq2, 4dst, 4dsu, 4duh, 4dv8, 4dy6, 4dzy, 4e0x, 4e1k, 4e3g, 4e4l, 4e4n, 4e5w, 4e67, 4e6d, 4e6q, 4e70, 4e7r, 4e9u, 4ea2, 4eb8, 4ef6, 4efk, 4efs, 4egk, 4ehz, 4ei4, 4ej8, 4ejl, 4ek9, 4elf, 4elg, 4elh, 4en4, 4eo6, 4eo8, 4eoh, 4eor, 4epy, 4er1, 4er2, 4erf, 4etz, 4eu0, 4euo, 4ew2, 4ew3, 4ewn, 4exs, 4ezx, 4ezz, 4f09, 4f0c, 4f1l, 4f2w, 4f39, 4f3c, 4f3k, 4f5y, 4f6u, 4f6w, 4f7v, 4f9u, 4f9w, 4f9y, 4fcq, 4fev, 4few, 4ffs, 4fht, 4fk6, 4fl1, 4fl2, 4flp, 4fm7, 4fm8, 4fnn, 4fp1, 4fs4, 4fsl, 4fxp, 4fys, 4fz3, 4fzj, 4g0p, 4g0q, 4g0y, 4g0z, 4g4p, 4g5f, 4g8m, 4g8n, 4g8v, 4g8y, 4g90, 4g95, 4gah, 4ge1, 4gfm, 4gfo, 4gg7, 4ggz, 4ghi, 4gid, 4gih, 4gii, 4gj2, 4gj3, 4gkh, 4gki, 4gkm, 4gny, 4gql, 4gqp, 4gqq, 4gqr, 4gr0, 4gr3, 4gr8, 4gu6, 4gu9, 4gue, 4gzp, 4gzt, 4gzw, 4gzx, 4h3f, 4h3g, 4h3j, 4h42, 4h75, 4h7q, 4h81, 4h85, 4ha5, 4hbm, 4hdp, 4heg, 4hfp, 4hge, 4hj2, 4hla, 4hp0, 4hpi, 4ht0, 4ht2, 4hu1, 4hw3, 4hwo, 4hwp, 4hws, 4hy1, 4hym, 4hzm, 4i3z, 4i54, 4i5c, 4i71, 4i72, 4i74, 4i7j, 4i7k, 4i7l, 4i7m, 4i7p, 4i8n, 4i8w, 4i8x, 4i8z, 4i9h, 4i9u, 4ibb, 4ibc, 4ibd, 4ibe, 4ibf, 4ibg, 4ibi, 4ibj, 4ibk, 4idn, 4ido, 4ieh, 4igt, 4ih5, 4ih6, 4ih7, 4iic, 4iid, 4iie, 4iif, 4ij1, 4in9, 4io2, 4io3, 4io4, 4io5, 4io6, 4io7, 4ipi, 4ipj, 4ipn, 4ish, 4isi, 4isu, 4itp, 4iue, 4iuo, 4iva, 4ivb, 4ivc, 4ivd, 4iwz, 4j21, 4j22, 4j28, 4j3l, 4j44, 4j45, 4j46, 4j47, 4j48, 4j7d, 4j7e, 4j93, 4jal, 4je7, 4je8, 4jfk, 4jfm, 4jfs, 4jh0, 4jia, 4jkw, 4jn2, 4jne, 4jpx, 4jpy, 4jsa, 4jss, 4jsz, 4jwk, 4jx9, 4jxs, 4jyb, 4jyc, 4jym, 4jyt, 4jz1, 4jzi, 4k0o, 4k0y, 4k18, 4k3h, 4k4j, 4k55, 4k5p, 4k6i, 4k77, 4k7i, 4k7n, 4k7o, 4k9y, 4kao, 4kax, 4kb9, 4kcx, 4kfq, 4kif, 4kiu, 4km0, 4km2, 4kmz, 4kn0, 4kn1, 4kni, 4knj, 4knm, 4knn, 4ko8, 4kow, 4kp5, 4kp8, 4kqp, 4ks1, 4ks4, 4ksy, 4kwf, 4kwg, 4kwo, 4kx8, 4kxb, 4kxn, 4kyk, 4kz3, 4kz4, 4kz6, 4kz7, 4kzq, 4kzu, 4l19, 4l2l, 4l4v, 4l4z, 4l50, 4l51, 4l6t, 4l9i, 4lar, 4lbu, 4leq, 4lhm, 4lhv, 4lj5, 4lj8, 4ljh, 4lk7, 4lkk, 4lko, 4lkq, 4ll3, 4llj, 4llk, 4llp, 4llx, 4lm0, 4lm1, 4lm2, 4lm3, 4lm4, 4loh, 4loi, 4loo, 4lov, 4loy, 4lps, 4lrr, 4luz, 4lvt, 4lxd, 4lxz, 4ly1, 4ly9, 4lyw, 4lzr, 4lzs, 4m0e, 4m0f, 4m0r, 4m0y, 4m12, 4m13, 4m14, 4m2r, 4m2u, 4m2v, 4m2w, 4m3p, 4m6u, 4m7j, 4m8e, 4m8h, 4m8x, 4m8y, 4mc1, 4mc2, 4mc6, 4mc9, 4mdn, 4mgd, 4mhy, 4mhz, 4mjp, 4mmm, 4mmp, 4mn3, 4mnp, 4mo4, 4mo8, 4mpn, 4mq6, 4mr3, 4mr6, 4mre, 4mrg, 4mrw, 4mrz, 4msa, 4msc, 4msn, 4mss, 4muf, 4mul, 4muv, 4myd, 4n07, 4n5d, 4n6g, 4n6z, 4n7u, 4n8q, 4n9a, 4n9c, 4na9, 4nbk, 4nbl, 4nbn, 4ncn, 4ndu, 4nh7, 4nh8, 4nj9, 4nja, 4nkt, 4nku, 4nl1, 4nnr, 4non, 4np2, 4np3, 4np9, 4nra, 4nuc, 4nue, 4nvp, 4nwc, 4nxu, 4nxv, 4nyf, 4o04, 4o05, 4o07, 4o09, 4o0a, 4o0b, 4o0x, 4o0y, 4o2b, 4o2c, 4o2p, 4o3c, 4o3f, 4o61, 4o97, 4o9v, 4o9w, 4oag, 4oak, 4ocq, 4oct, 4oeu, 4og3, 4og4, 4ogj, 4oiv, 4oks, 4oma, 4omc, 4omj, 4omk, 4or6, 4ovf, 4ovg, 4ovh, 4owm, 4owv, 4ozj, 4p3h, 4p58, 4p5d, 4p5z, 4p6c, 4p6w, 4p6x, 4pcs, 4pee, 4pf5, 4pft, 4pfu, 4pg9, 4phu, 4pin, 4pmm, 4pnu, 4poh, 4poj, 4pop, 4pow, 4pox, 4pp0, 4pp3, 4pp5, 4psb, 4pum, 4pvx, 4pvy, 4pzv, 4q08, 4q09, 4q0k, 4q19, 4q1w, 4q1x, 4q1y, 4q46, 4q4o, 4q4p, 4q4q, 4q4r, 4q4s, 4q6d, 4q6e, 4q7p, 4q7s, 4q7v, 4q7w, 4q81, 4q83, 4q87, 4q8y, 4q90, 4q93, 4q99, 4q9o, 4q9y, 4qac, 4qb3, 4qd6, 4qdk, 4qem, 4qer, 4qev, 4qew, 4qf7, 4qf8, 4qf9, 4qfl, 4qfn, 4qfo, 4qfp, 4qgd, 4qgi, 4qij, 4qj0, 4qjw, 4qjx, 4ql1, 4qlk, 4qll, 4qnb, 4qp2, 4qpd, 4qpl, 4qrh, 4qsu, 4qsv, 4qtl, 4qxo, 4qy3, 4qyy, 4r06, 4r0a, 4r3w, 4r4c, 4r4i, 4r4o, 4r4t, 4r59, 4r5a, 4r5b, 4r5t, 4r73, 4r74, 4r75, 4ra1, 4rak, 4rd0, 4rd3, 4rd6, 4rdn, 4re2, 4re4, 4rfc, 4rfd, 4rfm, 4rfr, 4rhx, 4riu, 4riv, 4rj8, 4rlt, 4rlu, 4rlw, 4rn4, 4rpn, 4rpo, 4rqk, 4rqv, 4rr6, 4rra, 4rrf, 4rrg, 4rsk, 4rux, 4ruy, 4ruz, 4rvr, 4rwj, 4rww, 4ryd, 4s1g, 4sga, 4std, 4tim, 4tjz, 4tkb, 4tkh, 4tkj, 4tln, 4tmk, 4tmn, 4tpw, 4tqn, 4trc, 4ts1, 4tt2, 4tte, 4tu4, 4tun, 4twp, 4ty6, 4ty7, 4tz2, 4u0f, 4u0w, 4u43, 4u54, 4u5n, 4u5o, 4u5s, 4u8w, 4ua8, 4uac, 4ual, 4uc5, 4ucc, 4ufh, 4ufi, 4ufj, 4ufk, 4ufl, 4ufm, 4uin, 4uj1, 4uj2, 4uja, 4ujb, 4und, 4unp, 4uof, 4uoh, 4ury, 4urz, 4us3, 4uye, 4uyf, 4v01, 4v24, 4v27, 4w52, 4w97, 4w9c, 4w9d, 4w9f, 4w9h, 4w9i, 4w9j, 4w9k, 4w9l, 4w9o, 4w9p, 4wa9, 4whs, 4wiv, 4wk1, 4wkb, 4wkn, 4wko, 4wkp, 4wn5, 4wop, 4wov, 4wrb, 4wt2, 4x24, 4x3k, 4x48, 4x50, 4x5p, 4x5q, 4x5r, 4x5y, 4x5z, 4x6m, 4x6n, 4x6o, 4x8o, 4x8u, 4x8v, 4xaq, 4xar, 4xas, 4xip, 4xiq, 4xir, 4xit, 4xk9, 4xmb, 4xmr, 4xo8, 4xoc, 4xoe, 4xt2, 4xtv, 4xtw, 4xtx, 4xty, 4xtz, 4xu0, 4xu1, 4xu2, 4xu3, 4xxh, 4xy8, 4xya, 4y0a, 4y2q, 4y3j, 4y3y, 4y4j, 4y59, 4y5d, 4y79, 4y8x, 4ybk, 4yc0, 4yes, 4ygf, 4yhm, 4yho, 4yk0, 4ykj, 4ykk, 4ymb, 4ymg, 4ymh, 4yml, 4ymq, 4ymx, 4ynb, 4ynl, 4yo8, 4yrd, 4ysl, 4ytc, 4yth, 4yx4, 4yxi, 4yyt, 4yzu, 4z07, 4z0k, 4z0q, 4z1e, 4z1j, 4z1k, 4z2b, 4z83, 4z84, 4z93, 4zae, 4zb6, 4zb8, 4zba, 4zbf, 4zbi, 4zeb, 4zec, 4zei, 4zek, 4zgk, 4zip, 4zji, 4zl4, 4zls, 4zme, 4zo5, 4zow, 4zt8, 4zv1, 4zv2, 4zvi, 4zw5, 4zw6, 4zw7, 4zw8, 4zwx, 4zwz, 4zx0, 4zx1, 4zx3, 4zx4, 4zyf, 4zzd, 4zzx, 4zzy, 4zzz, 5a2i, 5a5q, 5a6k, 5a6x, 5a7b, 5a7y, 5a81, 5aa9, 5aan, 5aba, 5acy, 5ad1, 5ahw, 5alb, 5am6, 5am7, 5amd, 5amg, 5aml, 5ant, 5anu, 5anv, 5aoi, 5aoj, 5aol, 5aqz, 5aut, 5ave, 5avf, 5ayt, 5azf, 5b2d, 5b5f, 5b5g, 5boj, 5bry, 5bs4, 5btv, 5btx, 5bw4, 5bwc, 5byi, 5c1m, 5c1w, 5c28, 5c2a, 5c2h, 5c2o, 5c3p, 5c5t, 5c8n, 5cap, 5caq, 5cas, 5cau, 5cbr, 5cbs, 5cc2, 5cep, 5ceq, 5chk, 5cj6, 5cjf, 5cks, 5cp5, 5cp9, 5cqt, 5cqu, 5cs3, 5cs6, 5cso, 5csp, 5cst, 5ct2, 5cu4, 5cxa, 5cy9, 5czm, 5d0c, 5d0r, 5d1r, 5d21, 5d24, 5d25, 5d26, 5d3c, 5d3h, 5d3j, 5d3l, 5d3n, 5d3p, 5d3t, 5d3x, 5d45, 5d47, 5d48, 5d6j, 5dbm, 5dex, 5dey, 5dfp, 5dgu, 5dgw, 5dh4, 5dh5, 5dhu, 5dit, 5dkn, 5dlx, 5dnu, 5dpx, 5dq8, 5dqc, 5dqe, 5dqf, 5dus, 5duw, 5dw2, 5dwr, 5dx4, 5dxt, 5dyo, 5e13, 5e1s, 5e28, 5e2k, 5e2l, 5e2o, 5e2p, 5e2r, 5e3a, 5e6o, 5e73, 5e74, 5e7n, 5e89, 5e8f, 5ect, 5edb, 5edc, 5edd, 5edl, 5ef7, 5efc, 5egm, 5egu, 5eh5, 5eh7, 5eh8, 5ehq, 5ehr, 5ehv, 5ehw, 5eis, 5ekm, 5en3, 5epl, 5epn, 5eq1, 5eqe, 5eqp, 5eqy, 5er1, 5er2, 5er4, 5etb, 5etj, 5eu1, 5ev8, 5evb, 5evd, 5evk, 5evz, 5ew0, 5ewa, 5ewk, 5ewy, 5exl, 5exm, 5exn, 5exw, 5ey0, 5ey4, 5eyr, 5f08, 5f0f, 5f1h, 5f1r, 5f1v, 5f1x, 5f25, 5f2p, 5f2r, 5f5z, 5f60, 5f61, 5f62, 5f63, 5f74, 5f8y, 5f9b, 5fbi, 5fck, 5fcz, 5fdc, 5fdi, 5fe6, 5fe7, 5fe9, 5fh8, 5fhm, 5fhn, 5fho, 5fl4, 5fl5, 5fl6, 5flo, 5flq, 5fls, 5flt, 5fnc, 5fnd, 5fnf, 5fng, 5fnr, 5fns, 5fnt, 5fnu, 5fog, 5fol, 5fot, 5fou, 5fov, 5fox, 5fpk, 5fs5, 5fsn, 5fso, 5fsx, 5fsy, 5fto, 5fut, 5fwr, 5fyx, 5g1z, 5g46, 5g4m, 5g4n, 5g4o, 5g5f, 5g5z, 5g60, 5g61, 5gj9, 5gja, 5gmh, 5gmn, 5gof, 5gs9, 5gsa, 5h1t, 5h1u, 5h1v, 5h5f, 5h8e, 5h8g, 5h9r, 5ha1, 5hbn, 5hbs, 5hct, 5hcv, 5hcy, 5hi7, 5hjq, 5hrv, 5hrw, 5hrx, 5htl, 5htz, 5hu9, 5hva, 5hvs, 5hvt, 5hwv, 5hz5, 5hz6, 5hz8, 5hz9, 5i1q, 5i29, 5i2e, 5i2f, 5i3a, 5i3v, 5i3w, 5i3x, 5i3y, 5i7x, 5i7y, 5i80, 5i88, 5i8g, 5i9x, 5i9y, 5i9z, 5ia0, 5ia1, 5ia2, 5ia3, 5ia4, 5ia5, 5ie1, 5igm, 5ih9, 5ihh, 5ii2, 5ikb, 5ime, 5ioz, 5ipc, 5ipj, 5irr, 5isz, 5ito, 5itp, 5ivc, 5ive, 5ivv, 5ivy, 5iwg, 5ix0, 5izf, 5izj, 5j0d, 5j1r, 5j3l, 5j41, 5j6a, 5j7q, 5j7w, 5j8z, 5ja0, 5jfp, 5jfu, 5jg1, 5jhb, 5ji8, 5jop, 5jox, 5jq5, 5js3, 5jsg, 5jsj, 5jsq, 5jss, 5jt9, 5jvi, 5jxn, 5jxq, 5jy3, 5jzi, 5k03, 5k0h, 5k1d, 5k1f, 5k8s, 5k9w, 5ka1, 5ka7, 5ka9, 5kab, 5kad, 5kat, 5kax, 5kbe, 5kby, 5kcb, 5kej, 5khm, 5kly, 5km9, 5kma, 5ko1, 5kqx, 5kqy, 5kr0, 5kr1, 5kr2, 5kva, 5kz0, 5l2s, 5l30, 5l3a, 5l4i, 5l4j, 5l4m, 5l7e, 5l7g, 5l7h, 5l8a, 5l9g, 5l9i, 5l9l, 5l9o, 5ld8, 5ldm, 5ldp, 5lif, 5ljq, 5ljt, 5lne, 5lom, 5lsg, 5lsh, 5lso, 5lud, 5lvd, 5lvl, 5lvq, 5lvr, 5lwd, 5lwm, 5lyn, 5lyr, 5lz4, 5lz5, 5lz7, 5m04, 5m17, 5m23, 5m25, 5m28, 5m5d, 5m77, 5m7s, 5m7u, 5m9w, 5ma7, 5meh, 5mek, 5mes, 5mg2, 5mge, 5mgf, 5mgj, 5mgk, 5mjn, 5mkr, 5mks, 5mn1, 5mnr, 5mo8, 5mod, 5mpz, 5mqe, 5mrb, 5mrm, 5mro, 5mxf, 5my8, 5mz8, 5n0d, 5n0e, 5n0f, 5n17, 5n18, 5n1r, 5n1s, 5n1z, 5n24, 5n25, 5n2t, 5n2z, 5n31, 5n34, 5n3v, 5n3y, 5n6s, 5n84, 5n93, 5n9r, 5nbw, 5ndf, 5ne5, 5nea, 5neb, 5nee, 5ngz, 5nih, 5njz, 5nk2, 5nk3, 5nk4, 5nk6, 5nk7, 5nk9, 5nka, 5nkb, 5nkc, 5nkd, 5nkg, 5nkh, 5nki, 5nn5, 5nn6, 5nvv, 5nvw, 5nvx, 5nw0, 5nw1, 5nw2, 5nwi, 5o07, 5o2d, 5o4f, 5o58, 5oei, 5oku, 5oot, 5op4, 5op5, 5oq8, 5orv, 5orw, 5os2, 5os4, 5os5, 5ose, 5ot8, 5ot9, 5ota, 5otc, 5ouh, 5ovr, 5ovx, 5std, 5sxm, 5sym, 5sz0, 5sz1, 5sz2, 5sz3, 5sz4, 5sz5, 5sz6, 5sz7, 5t19, 5t7s, 5t8o, 5t8p, 5t9u, 5t9w, 5t9z, 5ta2, 5ta4, 5tb6, 5tbe, 5tbm, 5tcj, 5tcy, 5tfx, 5th4, 5ti0, 5tmn, 5tmp, 5tp0, 5tpx, 5tt3, 5ttw, 5tuo, 5tuz, 5twj, 5txy, 5ty9, 5tya, 5u0d, 5u0e, 5u0f, 5u0g, 5u0w, 5u0y, 5u0z, 5u11, 5u12, 5u13, 5u14, 5u28, 5u49, 5u4b, 5u4d, 5u6j, 5u8c, 5ueu, 5uez, 5uf0, 5ufc, 5uff, 5ufp, 5ufr, 5ufs, 5uk8, 5ula, 5uln, 5ult, 5uoo, 5uov, 5upe, 5upf, 5upj, 5upz, 5ut6, 5uv2, 5uxf, 5v0n, 5v79, 5v7a, 5v82, 5vb5, 5vb6, 5vb7, 5vc3, 5vc4, 5vcv, 5vcw, 5vcy, 5vcz, 5vd0, 5vd1, 5vd2, 5vd3, 5vgy, 5vi6, 5vkc, 5vo1, 5voj, 5vp9, 5vsf, 5vsj, 5w1e, 5wa8, 5wa9, 5wal, 5wbm, 5wbo, 5wcm, 5we9, 5wex, 5wl0, 5wlo, 5wp5, 5wqc, 5wuk, 5wxh, 5wyx, 5wyz, 5x54, 5x62, 5x74, 5xg5, 5yas, 5yjm, 6ayi, 6b4l, 6b4n, 6b4u, 6b7a, 6b7b, 6en5, 6ep4, 6eqp, 6equ, 6euw, 6eux, 6ezq, 6rnt, 6std, 6upj, 7std, 7upj, 966c

**Table S1.** Atom features used in the graph representation of molecules.

| Feature | Description | Type | Size |
| --- | --- | --- | --- |
| Atom type | atom type | one-hot | 24 |
| Atomic number | atomic number | integer | 1 |
| Degree | the number of heavy atom neighbors (0 to 6) | one-hot | 7 |
| Number of hydrogens | the number of neighboring hydrogens (0 to 4) | one-hot | 5 |
| Implicit valence | the number of implicit hydrogens (0 to 6) | one-hot | 7 |
| Hybridization | *sp*, *sp^2^*, *sp^3^*, *sp^3^d*, or *sp^3^d^2^*. | one-hot | 5 |
| Formal charge | atomic formal charge (-3 to +3) | one-hot | 7 |
| Ring size | whether this atom belongs to a ring (ring size: 3 to 8) | binary | 6 |
| Aromaticity | whether this atom is part of an aromatic system. | binary | 1 |
| Acid/base | whether this atom is acidic or basic | binary | 2 |
| Hydrogen bonding | whether this atom is a hydrogen bond donor or acceptor | binary | 2 |
| Total |  |  | 67 |

**Table S2.** Hyperparameters explored for InteractionNet.

| Group | Hyperparameter | Size |
| --- | --- | --- |
| Graph Embedding | number of output units | 128, 256, 512 |
|  | number of embedding layers | 1, 2 |
| Graph Convolution | number of output units | 128, 256, 512 |
|  | number of convolution layers each | 0, 1, 2, 3 |
| Fully connected | number of output units | 128, 256, 512 |
|  | number of fully-connected layers | 2, 3 |
|  | l2 regularization | 0.0025, 0.005, 0.01, 0.02, 0.04 |
| Training | batch size | 32 |
|  | initial learning rate | 0.00015 |
|  | patience | 100, 200, 400 |
|  | loss | MSE |
|  | gradient descent method | Adam |


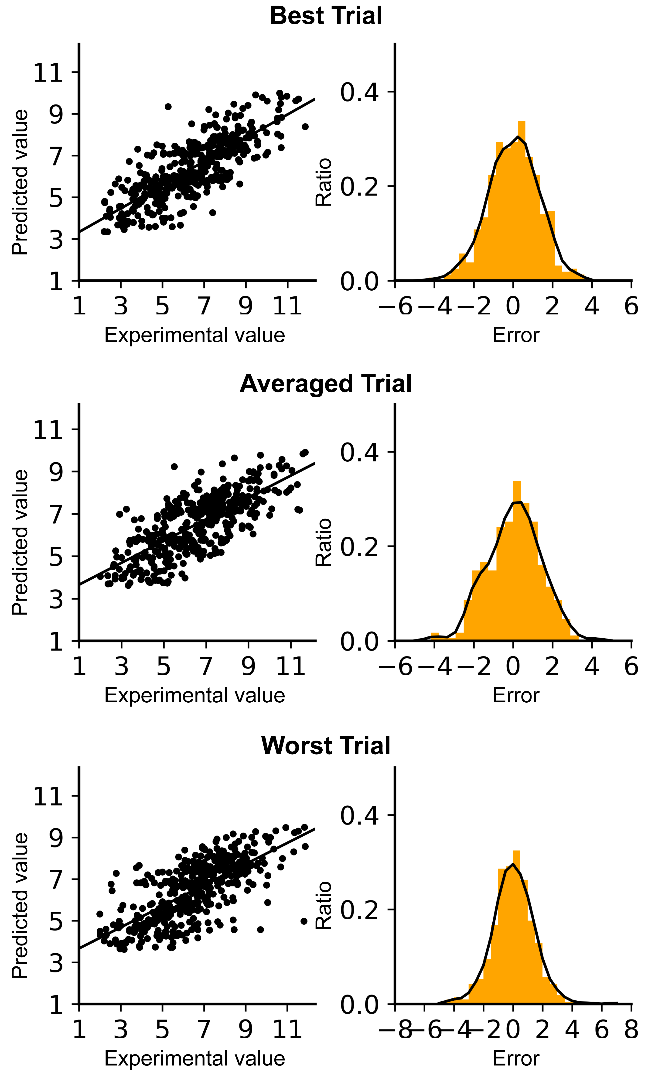


**Figure S1.** (Left) Scatterplots and (right) error distributions of predicted dissociation constants and experimental values, included in the test set across the best, averaged, and worst cross-validation trials. (Left) The scatterplot for predicted versus experimental constants is depicted with the solid trend line. (Right) The histogram (orange) and distribution (black) for predictions from the test set.
